# Understanding the Consequences of Fatty Bone and Fatty Muscle: How the Osteosarcopenic Adiposity Phenotype Uncovers the Deterioration of Body Composition

**DOI:** 10.3390/metabo13101056

**Published:** 2023-10-07

**Authors:** Kelsey Hu, Elizabeth Deya Edelen, Wenqing Zhuo, Aliya Khan, Josselyne Orbegoso, Lindsey Greenfield, Berna Rahi, Michael Griffin, Jasminka Z. Ilich, Owen J. Kelly

**Affiliations:** 1 Department of Molecular and Cellular Biology, Sam Houston State University College of Osteopathic Medicine, Conroe, TX 77304, USA; kyh004@shsu.edu (K.H.); ebd010@shsu.edu (E.D.E.); wzhuo@shsu.edu (W.Z.); axk084@shsu.edu (A.K.); jorbegoso@shsu.edu (J.O.); lcg028@shsu.edu (L.G.); mxg166@shsu.edu (M.G.); 2 Department of Human Sciences, Sam Houston State University College of Health Sciences, Huntsville, TX 77341, USA; bxa051@shsu.edu; 3 Institute for Successful Longevity, Florida State University, Tallahassee, FL 32304, USA; jasminkaernst@gmail.com

**Keywords:** osteoporosis, sarcopenia, obesity, adiposity, fat infiltration, osteopenic adiposity, sarcopenic adiposity, osteosarcopenic adiposity, osteosarcopenic obesity

## Abstract

Adiposity is central to aging and several chronic diseases. Adiposity encompasses not just the excess adipose tissue but also body fat redistribution, fat infiltration, hypertrophy of adipocytes, and the shifting of mesenchymal stem cell commitment to adipogenesis. Bone marrow adipose tissue expansion, inflammatory adipokines, and adipocyte-derived extracellular vesicles are central to the development of osteopenic adiposity. Adipose tissue infiltration and local adipogenesis within the muscle are critical in developing sarcopenic adiposity and subsequent poorer functional outcomes. Ultimately, osteosarcopenic adiposity syndrome is the result of all the processes noted above: fat infiltration and adipocyte expansion and redistribution within the bone, muscle, and adipose tissues, resulting in bone loss, muscle mass/strength loss, deteriorated adipose tissue, and subsequent functional decline. Increased fat tissue, typically referred to as obesity and expressed by body mass index (the latter often used inadequately), is now occurring in younger age groups, suggesting people will live longer with the negative effects of adiposity. This review discusses the role of adiposity in the deterioration of bone and muscle, as well as adipose tissue itself. It reveals how considering and including adiposity in the definition and diagnosis of osteopenic adiposity, sarcopenic adiposity, and osteosarcopenic adiposity will help in better understanding the pathophysiology of each and accelerate possible therapies and prevention approaches for both relatively healthy individuals or those with chronic disease.

## 1. Introduction

The well-recognized physiological hallmarks of aging related to body composition are loss of bone mass leading to osteopenia/osteoporosis, loss of muscle mass and strength leading to sarcopenia (referred here as the progressive loss of skeletal muscle mass) and dynapenia/myopenia (referred here as the progressive decline in skeletal muscle function/strength), respectively, and an increase in body fat, along with its redistribution and infiltration (to include local tissue adipogenesis from mesenchymal stem cell precursors commitment change and lipid accumulation in osteoblasts and myocytes) into other organs [1,2]. Ultimately, these changes lead to osteosarcopenic adiposity (OSA) [3], the most advanced stage of body composition deterioration, and the resulting negative consequences on other health outcomes [4]. Therefore, assessing body composition is an important clinical approach, not just in older individuals but throughout life. This is particularly important in view of growing evidence showing the role of body fat/adipose tissue (coupled with chronic inflammation) in the pathophysiology of numerous diseases [4,5], especially diabetes [6]. In particular, ectopic fat mass has negative effects on cardiometabolic health [7].

Obesity, defined by body mass index (BMI), is occurring at earlier ages than ever before [8]. The effects it has on bone and muscle health in younger individuals, especially in the long term, are of utmost importance and need investigation. One of the obstacles lies in the identification of overweight/obesity. BMI has served as the most common way for overweight/obesity diagnoses for decades, although obesity is a state of excess body fat [9], which BMI does not directly capture. Therefore, the term adiposity is used here to distinguish obesity (synonymous with BMI) from the effects of excess fat mass (overt and hidden) on the health status of the entire body, particularly musculoskeletal health. Despite BMI being a simple, quick, and inexpensive way to estimate overweight/obesity, BMI limitations have been recognized, particularly in the view that it might not be capturing the full picture of adiposity at the individual-patient level [9,10]. It becomes the most important encounter, if not a problem, for clinicians dealing with single patients at the individual level. The focus on BMI has created the phenomenon of normal-weight obesity, defined as having a normal BMI but a high body fat percentage (sometimes referred to as ‘skinny fat’) and carries the same risk profile as someone with a high BMI [9,10,11], estimated to affect 30 million Americans [10]. This clearly suggests that the focus should be on identifying adiposity, not just as increased body fat, but recognizing its heterogeneity in the form of excess/visible fat (subcutaneous), infiltrated fat, and redistributed fat (mostly visceral). None of this detail is indicated using BMI, nor does it distinguish between bone, muscle, and fat tissues. More importantly, BMI cannot indicate the true culprit of bone and muscle dysregulation, i.e., adipocyte infiltration and fat redistribution from the subcutaneous space to the visceral organs. Hypothetically, an individual, even at a younger age presenting with a normal BMI could insidiously experience bone loss (defined as a T-score at least one standard deviation below the standard for the population [12]) and/or sarcopenia (typically defined as the progressive loss of muscle mass, function and performance [13]) or osteosarcopenic adiposity (defined as the presence of bone and muscle loss within the context of adiposity) [14]. This review aims to lay the groundwork for incorporating the central role of fat mass (adipose tissue hypertrophy, redistribution, and infiltration) into the operational definitions of osteopenia/osteoporosis, sarcopenia, and obesity (adiposity). Thus, osteopenia/osteoporosis would be considered as ‘fatty bone’, sarcopenia would be considered ‘fatty muscle’, and adiposity would be considered an issue of excess fat and ectopic fat mass, leading to poor health consequences. This would also highlight the limitations of BMI in truly assessing fat mass and, consequently, truly assessing body composition.

Therefore, the aim of this narrative review is to re-establish excess adipose and malapropos adipose tissue in bone and muscle as central to osteosarcopenic adiposity (OSA). There is a concern that adiposity is occurring at earlier ages (childhood obesity). The overall negative consequences on health will be greater, and people will have to live longer with chronic conditions. Therefore, bone loss and muscle loss may begin at an earlier age due to chronic dysregulation. Specifically, this review will operationalize adiposity in relation to bone loss (osteopenic adiposity—low bone mass with infiltrated fat; OA), muscle mass, strength, and functional loss (sarcopenic adiposity – low muscle mass with infiltrated fat; SA), and bone and muscle loss combined (OSA) being the most detrimental case of body composition impairment. It is worth noting that the terms ‘obesity’ and ‘adiposity’ are sometimes used interchangeably in these syndromes [4], as is often the case in the literature reviewed.

## 2. Osteoporosis/Osteopenia, Sarcopenia, and Adiposity

### 2.1. Prevalence of Osteoporosis/Osteopenia, Sarcopenia, and Adiposity

The global prevalence of osteoporosis is 23% for women and 12% for men [15]. In many cases, a clinician may not diagnose osteoporosis until after a fracture has occurred [16]. Osteopenia, or low bone mass, is more prevalent than osteoporosis, 51% for women and 35% for men [17]. This is alarming, especially as it could be considered pre-osteoporosis. Osteopenia may be more common in males [18,19,20], although more data for males are needed. Sarcopenia has an estimated global prevalence of at least 10% [21]. The worldwide prevalence of overweight and obesity, measured using BMI, is now so high that they are considered global epidemics. The rates vary with age. For example, for 50–54-year-old individuals, it peaks at >50% for overweight and >25% for obesity [22].

### 2.2. Diagnosis of Osteoporosis/Osteopenia, Sarcopenia, and Adiposity and Ongoing Issues

The revised diagnostic criteria for bone loss, muscle loss, and adiposity combinations were recently published [12]; see Table 1 for a summary. In addition, functional criteria to assess the severity of bone and muscle loss and adiposity were developed [23].

Osteoporosis and osteopenia are diagnosed by measuring the bone mass using dual-energy X-ray absorptiometry (DXA) or bioelectrical impedance analysis (BIA). DXA remains the gold standard for diagnosis of low bone mass. However, osteoporosis is more a disease of trabecular bone than cortical bone. The trabecular bone score, derived from DXA data, has been put forward as an option, although this remains an estimate [24]. Microcomputed tomography (Micro-CT) may better measure osteoporosis as it can measure trabecular number and quality [25]; however, it is not yet feasible for routine clinical use. Higher-resolution micro-CT is available to assess trabecular bone [26], and this also has not transitioned to being a routine clinical tool.

Sarcopenia was first described in 1989 by Rosenberg [13] and was defined using an objective measure of muscle mass. More recent approaches emphasize handgrip strength or a combination of other physical performance measures (e.g., walking speed and knee extension) as screening tools [27], which seem more reflective of dynapenia. However, an assessment of muscle mass using either DXA, BIA, computed tomography (CT), or magnetic resonance imaging (MRI) would still be required to diagnose sarcopenia correctly. Therefore, there is some flexibility in confirming sarcopenia in relation to imaging techniques, unlike osteopenia/osteoporosis, for which diagnosis is solely used based on DXA measurements, where in the real world, not every clinic has the same instruments. The criteria proposed by the Sarcopenia Definitions and Outcomes Consortium seem to focus more on the functional aspects, possibly in the interests of using easy and quick methods in the clinician’s office [28]. Despite these shortcuts or variations in measurements, it is important to note that maintaining an objective measure for sarcopenia diagnosis may be more important than ever, as sarcopenia obtained an International Classification of Diseases (ICD) code in 2016 (M62.84), which has elevated its importance in healthcare. The clinical diagnosis of sarcopenia has the potential to mislead the clinician if it solely relies on functionality (e.g., handgrip strength) as a diagnostic tool.

Fat mass, both overt and ectopic, contributes to bone and muscle loss, and there is a great need to begin to measure where it is and how much of it there is throughout life. However, as more is learned about the root causes of bone and muscle loss and adiposity, better, more applicable diagnostic criteria will be needed, especially for early detection. Apart from the condition of normal weight obesity (which could be termed normal weight adiposity), the concept of metabolically healthy obesity/obese has highlighted more issues with BMI. Metabolically healthy obesity/obese (MHO) is a high BMI with serum triglycerides, cholesterol, blood pressure, and fasting blood glucose within the healthy range without medication [29]. MHO is also associated with insulin sensitivity [30] and lower visceral and liver fat [29]. This suggests that visceral/ectopic fat contributes more to type 2 diabetes than subcutaneous fat. In a three-year follow-up study of metabolically unhealthy obesity/obese (MUO) compared to MHO participants, it was found that a ≥5% loss of visceral fat in the MUO group was associated with a reversal of MUO to MHO [31]. However, in MHO individuals, the adipose tissue may remain healthy, with intact extracellular matrix (ECM) remodeling, no fibrosis, and good mitochondrial function; however, this needs to be elucidated. Current data show that MHO does not strictly have a bone protective effect [32] in those with a mean BMI of 43 kg/m^2^, although more research is needed in this area. Other data show that MHO has significantly decreased serum parathyroid hormone (PTH) and insulin levels while significantly higher levels of serum magnesium and osteocalcin [33]. Conversely, higher hip BMD was associated with MUO but not MHO in an Iranian cross-sectional study [34]. For sarcopenia, the results are mixed, although sarcopenia is associated with worse metabolic health [35]. On one hand, there may not be a protective effect of MHO, as the skeletal muscle index was not significantly higher compared to the MHO group [36]. Conversely, the prevalence of sarcopenia was significantly lower in the MHO group compared to the MUO group [37]. The data from MHO studies suggest that infiltrated and visceral fat are more important than subcutaneous fat in bone and muscle mass loss. The MHO phenotype may have ‘healthy’ adipose tissue and may have less infiltrated fat in bone and muscle. These data make a strong case for investigating adiposity in MHO compared to MUO, as this may help answer many questions related to the metabolic effects of fat mass location.

### 2.3. Osteosarcopenic Adiposity or Combinations of Osteopenia/Osteoporosis, Sarcopenia, and Adiposity

Bone loss, muscle loss, and increased fat mass (adiposity) are typically referred to as separate entities. However, an individual can simultaneously present with combinations of two or more conditions. Baumgartner first coined the term sarcopenic obesity in 2000 [38], which is referred to as sarcopenic adiposity here. Binkley and Buehringby originally proposed the term sarco-osteopenia in 2009 [39], which later assumed the term osteosarcopenia [40]. Another possible combination is osteopenic adiposity (previously osteopenic obesity), a state of low bone mass with excess fat mass [3]. Ilich and colleagues first described the triad of low bone and muscle mass with high fat mass as osteosarcopenic obesity [3]. The authors revised the name to osteosarcopenic adiposity to clarify that it relates not just to a high fat content or high BMI but also to the redistributed and infiltrated fat in bone and muscle [4]. The prevalence of sarcopenic adiposity is estimated at 10% globally [41] and osteosarcopenia at 5–37%, depending on the population [42]. Specific data are not available to estimate the prevalence of osteopenic adiposity; however, the prevalence estimates for osteopenia may act as a proxy. The earlier prevalence of osteosarcopenic adiposity was estimated at 7–10% [43] but varies widely depending on the population and measurement techniques for diagnosis. The prevalence could even reach a rate above 80%, as recently reviewed [44].

Diagnostic criteria were originally developed to separately assess bone health, muscle health, or adiposity. Only recently have diagnostic criteria been combined. The assumption is that they each hold true when used in combination; however, this needs to be evaluated. Each of these combined syndromes needs more research focus, with more accurate measurements of adipose tissue, including its infiltration/redistribution in various tissues and the consensus about the cutoff values.

## 3. Uncovering the True Role of Adiposity in Alterations of Body Composition

**Figure 1:**
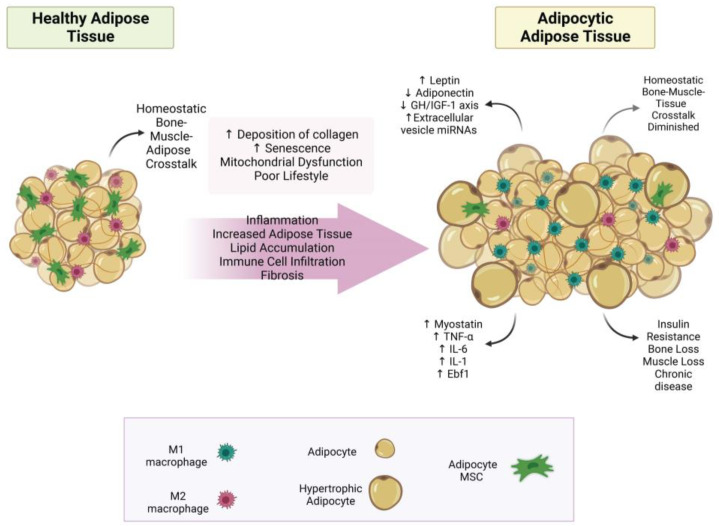
The transformation of adipose tissue to adipocytic adipose tissue. This figure summarizes how healthy adipose tissue develops into adipocytic adipose tissue. Abbreviations: (↓ = decreased; ↑ = increased; IL-6 = interleukin 6; IL-1 = interleukin 1; GH/IGF-1 axis = growth hormone/insulin-like growth factor-1 axis; Ebf1 = early B-cell factor 1; TNF-α = tumor necrosis factor-alpha; miRNA = micro RNA; MSC = mesenchymal stem cell. Created with BioRender.com.

The changes in adipose tissue as it transitions from a healthy state to a state of adiposity have been extensively reviewed, for example [45,46,47,48]; see Figure 1 for a summary. Briefly, adipocytes enlarge (hypertrophy) to store the surplus lipid. This stress on adipocytes within adipose tissue results in more inflammatory signals (adipokines) and increased recruitment of immune cells, especially with increases in M1 macrophages. These changes lead to systemic adipocytic inflammation, which causes adipose tissue expansion in other tissues, such as bone and muscle, and disruption of homeostatic bone–muscle–adipose cross-talk.

### 3.1. Bone–Muscle–Adipose Cross-Talk

#### 3.1.1. Bone–Adipose Cross-Talk

The reviews that have extensively examined the cross-talk between bone and adipose tissues have been published earlier (e.g., [3,49]). However, intriguing evidence suggests that adipocyte-derived extracellular vesicles may play significant roles in bone–adipose cross-talk. These vesicles typically contain proteins, lipids, and other molecules and serve as extracellular vehicles transporting circulating microRNAs (miRNAs). In bone, osteoblasts actively take up adipose tissue-derived extracellular vesicles, which deliver various miRNAs that affect metabolism [50]. One group has recently linked increases in specific miRNAs (miR-122, miR-192, miR-27a-3p, and miR-27b-3p) to central obesity in mice [51]. In addition, Zhang et al. found several white adipose tissue-derived extracellular vesicle miRNAs to be higher in obesity and to inhibit bone formation (promote osteoclastogenesis), clearly linking obesity/adiposity and bone loss [50]. While all data do not fully agree on the mechanisms of bone–adipose cross-talk [52], adipose tissue-derived extracellular vesicle uptake via osteoblasts provides strong evidence that adipose tissue talks to bone and suggests that the composition of vesicles may change as healthy adipose tissue transitions to adipocytic adipose tissue (See Figure 1). It also hypothesizes that bone and muscle may produce extracellular vesicle miRNAs to facilitate cross-talk.

#### 3.1.2. Muscle–Adipose Cross-Talk

Several groups have extensively reviewed the cross-talk between muscle and adipose tissue [3,49,53,54,55]. Cross-talk occurs via adipokines, myokines, and the adipo-myokines (molecules produced by both adipocytes and myocytes) such as interleukin-6 (IL-6) and tumor necrosis factor-α (TNF-α) [56]. The myokines, IL-6, irisin, insulin-like growth factor-1 (IGF-1), brain-derived neurotrophic factor (BDNF), myostatin, and fibroblast growth factor 2 (FGF2) have both catabolic and anabolic effects depending on their relative ratios, location, and physiological state. For example, aerobic activity has an anti-inflammatory effect via myocyte interleukin-10 (IL-10) and interleukin-1 receptor antagonist (IL-1-RA), both locally and, more importantly, systemically, suggesting it may contribute to modulating adipose tissue homeostasis [57]. Myostatin, a myokine, is a negative modulator of muscle mass; therefore, knockout animals have greatly elevated muscle mass [58]. However, low levels of myostatin may also inhibit adipose tissue expansion [57]. Exercise (muscle use) reduces myostatin locally and systemically [58], suggesting a sedentary lifestyle enhances myostatin from myokines, thereby decreasing muscle mass and increasing fat mass. Other myokines expressed after exercise, such as irisin and meteorin-like, may help induce the browning of white adipose tissue adipocytes [59]. This suggests exercise may control or reduce fat infiltration into muscle; however, there is much to learn about muscle–adipose cross-talk in normal-weight individuals compared to those with adiposity.

#### 3.1.3. Bone–Muscle Cross-Talk

Numerous reviews examining bone–muscle cross-talk are already available; for examples, see [49,60,61]. Both mechanical and biochemical signals connect muscle to bone [3,62]; these observations have been comprehensively reviewed [63]. Muscle paralysis, atrophy, or immobilization promote bone loss and osteoporosis [64]. In healthy individuals, targeted physical activity may improve bone–muscle cross-talk [62], reinforcing the concept that bone and muscle work together by design. The shared central pathway is the growth hormone/insulin-like growth factor-1 (GH/IGF-1) axis. This pathway plays a crucial role in regulating bone and muscle growth [65], and any factor that perturbs this system, including inflammation, a sedentary lifestyle, or a poor diet, can result in bone and muscle mass loss. Furthermore, sophisticated bidirectional cross-talk between muscle and bone, consisting of mechanical interaction and paracrine and endocrine communication, plays a role in bone and muscle homeostasis. Decreased muscle function and performance result in decreased skeleton load and subsequent deterioration of bone density.

### 3.2. Adipokines—Old and New

Adipose tissue functions as an essential endocrine organ critical for overall health and metabolic homeostasis, but its balance is crucial [66]. Two of the most notable adipokines influencing the development of OSA include adiponectin and leptin. However, adipose tissue influences bone and muscle via a multitude of adipokines. Apart from adiponectin and leptin, the sections below will briefly discuss some other potential adipokines in bone and muscle mass maintenance and/or loss. 

#### 3.2.1. Adiponectin

Serum concentrations of adiponectin, an adipokine, have been shown to decrease with age and obesity and have a strong negative relationship with fat mass [67]. Adiponectin signals through adiponectin 1 and 2 receptors (adipoR1 and adipoR2), which are abundantly expressed by osteoblasts, osteoclasts [68], and myocytes [69]. In addition, bone marrow adipose tissue (BMAT) produces adiponectin, indicating bone as a local source [68]. To our knowledge, there are no data on how much adiponectin is produced by inter- and intra-muscular adipose tissue. However, this local source in muscle may compensate for the reduced systemic levels seen in aging and obesity [70] and in sarcopenic adiposity [71]. The major roles of adiponectin in bone and muscle are to promote bone formation [68] and stimulate myogenesis [72], respectively. 

Adiponectin stimulates bone formation through binding to adipoR1, which leads to the phosphorylation of p38 mitogen-activated protein kinases (P38 MAPK), increasing cyclooxygenase-2 (Cox-2) and bone morphogenetic protein-2 (BMP-2) expression [66]. BMP-2 is highly osteogenic; however, although Cox-2 is considered inflammatory, prostaglandin E_2_ (PGE_2_), its product, has been shown to stimulate bone formation if the concentration is low [73]. In an obese state, there are increased local inflammatory cytokines (adipocytes and immune cells) such as IL-6, TNF-α, and systemic C-reactive protein (CRP) from the liver, which inhibit the secretion of adiponectin by adipocytes [74]. Conversely, some in vitro data suggest that adiponectin (presumably local and systemic sources) stimulates the receptor activator of the nuclear factor kappa-Β ligand (RANKL) pathway via adipoR1—P38 MAPK, thereby stimulating osteoclastogenesis [75]. However, osteoblasts produce RANKL, which is one of the first steps in building bone. Therefore, the presence or absence of additional bone turnover mediators may significantly modulate adiponectin signaling in bone, resulting in its dual effects in bone formation/resorption processes. The local production of adiponectin from BMAT needs to be investigated.

Regarding muscle, adiponectin plays a critical role in muscle mass growth and maintenance [76], and additional studies of adiponectin would help to understand how lower levels lead to muscle mass loss in aging. In addition, adipoR1 knockout mice present with significantly decreased type I (slow-twitch) muscle fibers [77], suggesting that adiponectin helps maintain these fibers. However, disagreement about whether higher or lower levels of adiponectin correlate with muscle loss in aging persists [76], indicating its nuanced role in maintaining muscle mass that may also depend on the amount produced locally by infiltrated adipose tissue. In muscle, 5′ adenosine monophosphate-activated protein kinase (AMPK), the master regulator of energy homeostasis and peroxisome proliferator-activated receptor α (PPARα), mediate most of the effects of adiponectin; PPARα also directly regulates the expression of enzymes involved in β-oxidation [70]. These data indicate that adiponectin may be essential for type I muscle fatty acid metabolism, especially in adiposity. This is interesting as type I fibers seem to be maintained in sarcopenic adiposity at the expense of type II muscle fibers (fast-twitch), indicating more investigation of its role in muscle fiber type loss in OSA is essential. 

#### 3.2.2. Leptin

Leptin, another adipokine, has a primary function in maintaining energy homeostasis [78]. The leptin concentration typically reflects the number of adipocytes; higher leptin levels correlate with more adipocytes. However, it is no surprise that this does not correlate well with BMI [79]. By increasing the production of TNF-α, IL-6, and IL-12 in monocytes, leptin also promotes systemic and local inflammation [80]. Osteoblasts, as do chondrocytes (involved in bone formation and fracture repair), express the leptin receptor, and locally BMAT secretes leptin [79]. Leptin promotes increased osteoblastic activity and decreased osteoclast activity, suggesting it is bone-friendly [66] or has a dual role. This agrees with the observation that leptin resistance in obesity (per BMI) is associated with worse bone outcomes [79]. However, indirectly, leptin also negatively impacts bone via hypothalamic signaling, as shown in animal studies [79], although this effect has not yet been established in human studies [81].

Muscle adipocytes avidly express leptin receptors, and a lack of leptin-sensitive receptors causes muscle atrophy [82]. Therefore, leptin resistance may worsen muscle mass loss in obese and elderly patients [83]. One group has reported that, by counteracting the inhibition of satellite cells in a miR-489-dependent process, leptin increases muscle mass and aids in the repair of muscle cells in animal models [84]. In aging mice, leptin administration inhibits the expression of myostatin, thereby stopping muscle decline [85]. Through activating AMPK, leptin also increases the oxidation of fatty acids, thereby reducing muscle lipid content [86]. Leptin inhibits appetite (signaling sufficient energy intake) and directly regulates bone and muscle mass via hypothalamic signaling [87]. This finding indicates that bone formation and muscle growth are sensitive to the overall energy state. At its most basic level, this suggests that in times of surplus energy (i.e., postprandial state), bone and muscle growth should be increased; however, this does not seem to occur in adiposity (which indicates surplus stored energy). Leptin resistance likely contributes significantly to OSA. As muscle cells cannot recognize or respond to leptin appropriately, the cells atrophy [84] despite their very high serum levels. 

#### 3.2.3. Chemerin

Chemerin is a relatively new adipokine involved in inflammation, adipogenesis, angiogenesis, and energy metabolism and may be a key adiposity-driving factor. It is expressed at high levels in white adipose tissue adipocytes and low levels in brown adipose tissue adipocytes. However, chemerin is expressed in many other tissues in humans, such as the liver, pancreas, adrenal gland, heart, lung, and ileum, as well as pericoronary and periaortic adipose tissue and epithelial cells of various vessels [88]. Within adipose tissue, the primary chemerin receptor, chemokine-like receptor 1 (Cmklr1), is expressed both by adipocytes and immune cells (immature dendrites, macrophages, and monocytes) and is responsible for chemotaxis, thereby linking adiposity and immune-mediated inflammation [89]. In humans, Cmklr1 is also expressed in the tissues that express chemerin and the testis and lymph nodes; its other receptor, chemerin receptor 2, seems to be universally expressed in humans [88]. This suggests that chemerin signaling is important in homeostatic whole-body cross-talk. However, the signaling mechanism is more complicated. Chemerin knock-out models (animal and cell) point to the mechanism being dependent on an increase in osteoclast and a decrease in osteoblast proliferation and differentiation, effectively modulating bone remodeling [90]. Conversely, while serum levels of chemerin are increased in obese mice, levels may not be increased in the bone marrow microenvironment, so there is no negative effect on bone mass [91]. In 543 Chinese obese postmenopausal women, serum chemerin levels were negatively correlated with bone mineral density after controlling for age, lean mass, and fat mass. The authors hypothesize that chemerin mediates its effects by inciting chronic low-grade inflammation [92]. Similarly, in 111 Chinese postmenopausal women who had a fracture, high serum chemerin levels were negatively associated with BMD and fracture risk [93]. In the same way, the data related to Cmklr1 knock-out models are raising more questions. Cmklr1^-/-^ mice present with lower serum testosterone levels and reduced trabecular bone mass. In addition, the inactivation of Cmklr1 in male wild-type mice led to lower bone mass [94]. Cmklr1 has been detected in the testes, which provides a mechanism for bone loss via lower serum testosterone [88], although more work is needed. Both chemerin and Cmklr1 are required to shift bone marrow stromal cells to the adipocyte lineage. Conversely, knockdown of Cmklr1 in bone marrow stromal cells, which were then treated with adipogenic stimuli, preserved osteoblast gene expression [95]. Interestingly, Cmklr1 is expressed in the parathyroid gland [96], suggesting that chemerin may lower bone mass via parathyroid hormone. Evidence suggests that chemerin plays a role in sarcopenia adiposity via disrupting mitochondrial function [97] and inflammatory mechanisms [98,99].

Chemerin may be a key adipokine in whole-body homeostasis due to its expression and its receptor expression in various tissues. Chemerin requires Cmklr1 to modulate bone metabolism. In general, cumulative data in humans suggest that chemerin and Cmklr1 act on bone via different mechanisms, although with the same loss of bone outcome. However, excess chemerin levels may be more important in relation to this discussion, as disease-causing Cmklr1 gene variants have not been detected in humans. Chemerin and Cmklr1 have an interesting future ahead.

#### 3.2.4. Early B-Cell Factor-1 (Ebf1)

A gene that may play a critical role in osteopenic adiposity is the transcription factor, Early B-cell factor-1 (Ebf1). It is highly expressed in adipose tissue adipocytes and plays a critical role in adipocyte differentiation [100,101]. The subsequent observation that global *Ebf1^−^^/^^−^* mice present with severe lipodystrophy, in addition to stunted growth and runting, confirms a crucial role for Ebf1in adipogenesis [101]. A perplexing observation reported in that same manuscript was that the *Ebf1^−^^/^^−^* mice also presented with a peculiar bone phenotype, the bone marrow filled up with ‘ghost adipocytes’, although the osteoblast number was also increased. *Ebf1^−^^/^^−^* mice also present significantly reduced circulating leptin levels, consistent with lipodystrophy, and increased food intake [102]. Low leptin levels are also associated with obesity. What makes the *Ebf1^−^^/^^−^* bone phenotype so interesting is the fact that a majority of human patients with anorexia nervosa (at least in one genome-wide association study of 700 females of European descent) have SNPs in *EBF1* and also have elevated levels of bone marrow adiposity and low leptin levels [103]. 

Ebf1 may reciprocally regulate adipogenesis and osteogenesis, commonly referred to as the ‘bone-fat switch’, through interactions with zinc finger proteins (Zfp) 423 and 521 [104,105,106]. Huo et al. demonstrated that expression of *Ebf1* in human white adipocytes negatively correlates with their size, i.e., smaller adipocytes have increased levels of Ebf1 [107]. One possibility (reviewed here [108]) is that Ebf1 is called into action quickly during an inflammatory stimulus in adipocytes, binds to and stimulates transcription of its targets, but then is quickly disposed of. Ebf1 promotes inflammation in adipocytes, suggesting that Ebf1 actively participates in the development of osteopenic adiposity.

### 3.3. Fat Infiltration in Bone and Muscle Loss

Increased localized adipose tissue deposits in bone and muscle are associated with bone and muscle loss [109]. Obesity (defined using BMI) has been historically perceived as protective against fractures; however, recent studies have suggested that this may not be the case [110], and there seems to be a threshold above which adiposity becomes detrimental to bone [111]. Therefore, a U-shaped curve better describes the relationship between osteoporosis and obesity [110]. Such a relationship has also been confirmed just recently in the analysis of 11,615 adults from NHANES data spanning from 1999 to 2018 [112]. The analysis showed the U-shaped curve (particularly in men) and the negative relationship between fat (total, visceral, android, and gynoid) and BMD. Other available data show that infiltrated fat is involved in bone deterioration [113,114,115,116] and supports some common mechanisms contributing to low bone mass and higher fat mass, such as inflammation [117], increased bone marrow adiposity [118,119,120], and senescence of osteoblastic progenitor cells [121,122,123]. From an endocrine perspective, there are clear links between increased adiposity and bone loss: increased levels of adiposity reduce growth hormone (GH) production, leading to higher visceral and total body fat accumulation and reduced lipolysis [124] and, consequently, bone loss [125]. A greater understanding of molecular mechanisms will increase opportunities for identifying therapeutic targets that can further minimize osteoporotic fracture risks in relation to higher adiposity and synergistically target both adiposity and bone health. Advances in technology to detect infiltrated fat in bone and muscle will be paramount in our understanding of these relationships.

A critical mechanism of bone loss involves the expansion of BMAT from bone marrow stromal cells (BMSCs) at the cost of osteoblasts. This ‘fattening’ of bone, coupled with reduced bone formation, decreases trabecular bone density, resulting in a higher risk for fractures [126]. BMAT expansion may also decrease hematopoiesis, contributing to anemia [127] and precipitating dynapenia [128]; more work in this area is warranted. It is more encouraging that BMAT can be measured noninvasively in humans using magnetic resonance spectroscopy (MRS) or magnetic resonance imaging (MRI) [129]. The broader issue is that measuring BMAT is highly specialized and is not quick, easy, and available in clinicians’ offices. BMAT increases with age and adiposity but does not decrease with lower energy intake and may even increase during periods of starvation or anorexia [130]. This suggests that BMAT may serve as an energy reserve for maintaining erythropoiesis during periods of nutritional stress. However, animal data suggest that BMAT responds to diet and exercise. In female C57BL/6 mice, a high-fat diet resulted in almost a 3-fold higher BMAT than in controls, which could be offset by higher physical activity [131], demonstrating that BMAT—like other types of adipose tissue—accumulates in the presence of excess energy and a sedentary state (Western lifestyle), just like obesity. 

During aging, adipose inflammation leads to fat redistribution from the subcutaneous space to the intra-abdominal fat (visceral fat), as well as in skeletal muscle (fat infiltration), resulting in decreased strength and muscle functionality [1]. As lipids accumulate within and between muscle cells, they precipitate myriad altered functions, including mitochondrial dysfunction, disrupted beta-oxidation of fatty acids, reactive oxygen species production, enhancement of pro-inflammatory cytokine secretion [132], and insulin resistance [133]. Intramuscular adipose tissue likely plays an essential and causal role in developing sarcopenia and muscle weakness. Muscle adiposity is associated with higher BMI even in younger (mean age 14 years) males and females [134], and infiltrated fat in the muscle is associated with accelerated loss of muscle mass in aging [135]. Fat infiltration of skeletal muscle, also referred to as myosteatosis, is gaining recognition for its role in muscle mass, strength, and function loss. In 2018, the National Institute on Aging held a workshop to deliberate on myosteatosis in the context of skeletal muscle function deficit. However, measuring fat infiltration (sometimes referred to as muscle quality) in muscle is complex, as intermuscular adipose tissue, intramuscular adipose tissue, and intramyocellular lipids are all included in the umbrella term myosteatosis [136]. In Rosenberg’s original 1997 paper [137], the figure comparing the thighs of younger and older women clearly illustrates the vast differences in muscle mass in sarcopenia. More importantly, Rosenberg also states that a ‘great deal more fat’ is present. While much of the research since then has focused on the loss of muscle mass, and deservedly so, there is clearly a central role for fat in the pathophysiology of muscle mass, strength and function loss. 

### 3.4. Other Aspects of Adiposity in Bone and Muscle

#### 3.4.1. Mitochondrial Dysfunction

One key function of bone–muscle–adipose cross-talk is whole-body energy balance [138], as all three are recognized as endocrine organs. The key organelle in cellular energy metabolism is the mitochondria. Adiposity (obesity) results in reduced mitochondrial function through various mechanisms, including increased oxidative stress and reduced mitochondrial biogenesis, and has been extensively reviewed, for example [139,140]. Standard treatments for obesity include dietary restrictions (similar to calorie restriction) and, in certain cases, bariatric surgery. Calorie restriction results in a down regulation of mitochondrial respiration rate, whereas bariatric did not, even though weight loss occurred in both situations [141]. The stress associated with major surgery and substantial changes to the gastrointestinal tract (Roux-en-Y gastric bypass surgery) may contribute to this difference. However, although Roux-en-Y gastric bypass surgery decreases fat mass, there is a lean mass loss for most patients, even with exercise interventions [142]; other procedures (gastric bypass, biliopancreatic diversion with the duodenal switch adjustable gastric band, and sleeve gastrectomy) have similar effects on the percent of fat mass loss [143]. A study using air displacement plethysmography to assess body composition before and twelve months after bariatric surgery showed that while the percent of lean body mass increased after surgery (from 45% to 62%), the average absolute loss of lean mass was 6.75 kg [144]. Bariatric surgery does result in more body fat loss compared to calorie restriction and exercise; however, bariatric surgery was not as effective as calorie restriction and exercise at reducing epicardial adipose tissue [145], which is strongly associated with cardiovascular risk [146]. This suggests that the adipose tissue repositories affected by exercise and calorie restriction differ from those affected by bariatric surgery. Considering the strong evidence that adipose tissue communicates with other tissues, changes to different fat depots may affect mitochondria differently in other tissues as well as adipose tissue.

However, as bone and muscle become fatter in adiposity, adipose tissue may negatively affect bone and muscle mitochondrial function. Mouse models demonstrate that aging results in mitochondrial DNA damage, thus reducing the capacity of osteoblasts to form bone [147]. Mitochondrial DNA damage may also be due to adiposity [148]. Oxidative stress and reduced mitochondrial biogenesis and mitophagy are also implicated in mechanisms of mitochondrial dysfunction in bone loss (osteoporosis) [149,150]. Similarly, poor mitochondrial quality control contributes to sarcopenia and has been extensively reviewed elsewhere, for example [151,152,153,154]. Mitochondrial dysfunction clearly contributes to bone loss, muscle loss, and adiposity. Nevertheless, what is not known is if infiltrated fat modulates osteoblast/osteocyte and myocyte mitochondrial function, it ultimately contributes to bone and muscle mass loss.

#### 3.4.2. Fibrosis

Fibrosis is typically thought of in the context of certain diseases, such as liver disease or scar tissue; however, in adiposity, the fibrosis process is initiated, resulting in the overproduction of extracellular matrix (ECM) proteins (predominately collagens) and other compounds. The topic has been reviewed previously, for example [155,156,157]. Adipocytic fibrosis results in less ECM remodeling compared to healthy adipose tissue, so ECM builds up, reducing the capacity of adipose tissue and immune cells to resolve inflammatory events and respond to adipose tissue expansion during excess dietary energy intake [157]. Fibrosis is usually not detected in adipose tissue in obesity, is resistant to treatment, and interferes with adipose tissue homeostasis, such as reducing lipolysis [155]. This results in more visceral fat depots, which may also be linked to the lower lipolysis in fibrotic adipose tissue [156], so new fat depots are needed. 

ECM is ubiquitous and is necessary for normal bone metabolism. Within bone, ECM facilitates cell motility, cell adhesion, proliferation and differentiation of bone cells, and the bone remodeling process, as well as being the foundation for mineralized bone itself (predominately collagen type I) [158]. Therefore, any disruption in bone ECM formation and remodeling can result in bone loss. In osteoporosis, changes to the ECM probably do occur, mediated by variances in osteocalcin (decreased), osteonectin (decreased), and osteopontin (increased), modulators of bone ECM [159]. It is intriguing to hypothesize that higher BMAT results in negative changes to the bone ECM; however, more research is needed. Similarly, homeostasis of ECM is needed to maintain muscle mass [160]. Changes in the ECM may be related to mitochondrial dysregulation in sarcopenia. The ECM (three layers: epimysium, perimysium, and endomysium (AKA the basement membrane)) can account for 10% of skeletal muscle weight and facilitate muscle function, homeostasis, and regeneration. However, in sarcopenia, more ECM is laid down (predominately composed of collagens), and increased collagen crosslinks lead to stiffness [161], probably decreasing muscle strength and functionality. All these data suggest that fibrosis, or dysregulated ECM, plays a role in adiposity and bone and muscle mass loss.

#### 3.4.3. Immune System

The immune system’s involvement in the adipose dysfunction and inflammation of obesity is well known and has been extensively reviewed; for example [162,163,164], leptin may be a key player in the adipose-immune system interactions [165]. However, the immune system is ubiquitous in tissues and organs; therefore, the role of the immune system in bone loss and muscle loss is becoming clearer. Terms such as ‘osteoimmunology’ and ‘immunoporosis’ are used to describe the field of research related to bone–immune connections. For example, several recent, excellent, in-depth reviews are available on the topic [166,167,168]. Bone cells and immune cells share common cytokines (osteokines and immunokines), and the key link between bone and the immune system is the receptor activator of NF-kB/receptor activator of NF-kB ligand/osteoprotegerin (RANK/RANKL/OPG) axis [168]. RANK/RNAKL binding is required for osteoclast differentiation; conversely, RANKL/OPG binding inhibits osteoclast differentiation [166]. The immune system has a role in several types of bone loss, including postmenopausal osteoporosis (estrogen may not directly affect bone mass; rather, estrogen depletion may work through the innate and adaptive immune system to reduce bone mass), senile osteoporosis (senescent macrophages and neutrophils promote bone resorption) and diabetes osteoporosis (hyperglycemia results in the polarization of M1 macrophages and non-polarization of M2 macrophages resulting in bone loss) [167]. RANKL (from osteoblasts) binding to the RANK receptor (osteoclasts) is the key pathway regulating the bone remodeling cycle. However, RANKL is also overexpressed by B- and T-lymphocytes and invariant natural killer cells in states of bone loss [168]. Inflammation is another important mechanism in osteoimmunology. Innate immune cells (macrophages and dendritic cells) and osteoclasts share a common stem cell ancestry and are considered inflammatory, as are granulocytes, innate lymphoid cells, and natural killer cells [166]. However, it is not that simple; others have found that CD8^+^ T-cells help form bone [169]. There is much to learn about the role of immune cells in bone loss and how BMAT interacts.

Sarcopenia could be considered a chronic site of injury in muscle [170]; in this sense, the immune system must play a role in the development of sarcopenia. In aged muscle, there is a pronounced shift to the M1 macrophage type (immune aging). As the M2 macrophage (increased by regulatory T-cells) is associated with activating satellite cells, this suggests a reduction in skeletal muscle repair and possibly chronic inflammation by M1 macrophages [171]. The interleukins 6, 7, and 15 modulate the immune system, and interleukin-7 and interleukin-15 are inversely associated with age [172]. A decrease in migrating neutrophils may also contribute to the lower level of muscle regeneration in older muscles [173]. There is a clear link between muscle metabolism and the immune system in aging [172]; however, less is known about changes to muscle induced by fat infiltration at younger ages.

## 4. Uncovering the Deterioration of Body Composition—Clinical Aspects

The effects of adiposity on bone, muscle, and adipose tissue have been discussed. Adipose tissue talks to bone and muscle via adipokines and adipose tissue-derived extracellular vesicle miRNAs. The latter offer an innovative opportunity for treatment (drug delivery) and possibly prevention strategies. Low-grade chronic inflammation represents the probable primary mechanism driving negative bone and muscle metabolism changes in the adipocytic state [5,174]. BMAT expansion likely complements this, as does the additional adipose tissue in the muscle, guaranteeing inflammation is not resolved. Bone–muscle–adipose cross-talk is necessary for homeostasis, and the resultant changes in adipocytic adipocytes probably disrupt or even stop these interactions. However, while this manuscript focuses on the role of adiposity in bone and muscle loss, hypothetically, bone–muscle–adipose cross-talk could be disrupted by either dysfunctional bone or muscle metabolism via osteokines and myokines, respectively. This section will provide the foundation for operationalizing the concept of adiposity into the clinical conditions of osteopenia/osteoporosis, sarcopenia, and obesity.

### 4.1. Osteopenic Adiposity 

**Figure 2:**
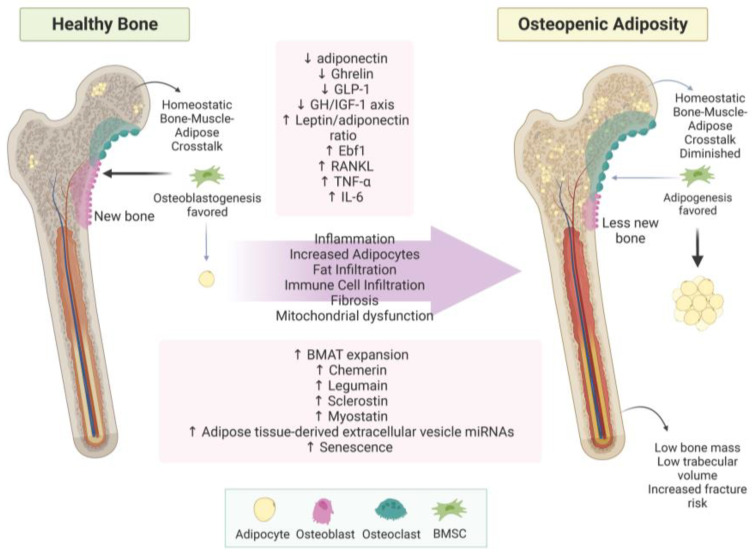
Osteopenic adiposity syndrome. This figure summarizes how healthy bone develops into osteopenic adiposity syndrome. Increased BMAT and fat infiltration are represented by yellow circles within the bone. Abbreviations: (↓ = decreased; ↑ = increased; BMSC = bone marrow stem cell; GLP-1 = glucagon-like peptide-1; GH/IGF-1 axis = growth hormone/insulin-like growth factor-1 axis; Ebf1 = early B-cell factor 1; RANKL = receptor activator of NF-κB (RANK) ligand; TNF-α = tumor necrosis factor-alpha; IL-6 = interleukin 6; BMAT = bone marrow adipose tissue; miRNA = micro RNA. Created with BioRender.com.

Osteopenic adiposity (OA) is a condition of fat infiltration into bones, with or without overtly high body fat mass, coupled with bone loss. Inflammation is a key contributor to OA and has been extensively reviewed previously; for examples, see [114,175,176,177]. Disruptions in bone–adipose cross-talk likely contribute; however, more research into osteopenic adiposity is warranted as consequences of bone loss represent a large healthcare burden, especially related to adipocyte-derived extracellular vesicles. OA processes are summarized in Figure 2.

### 4.2. Sarcopenic Adiposity

**Figure 3:**
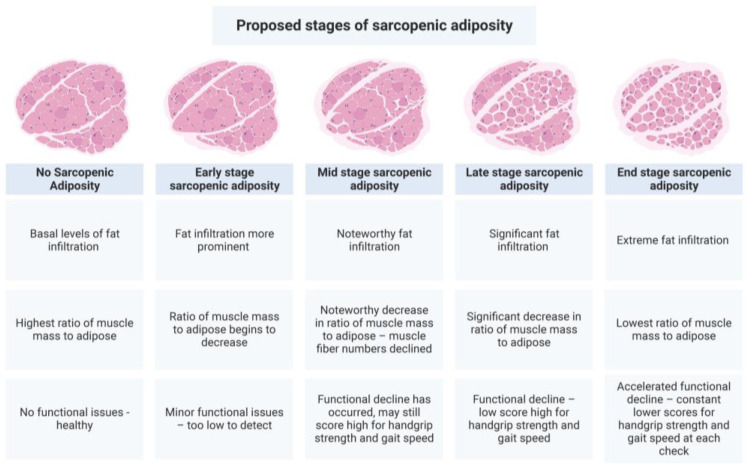
Proposed developmental stages of sarcopenic adiposity. This figure summarizes the concept that fat infiltration into muscle, in combination with muscle mass loss, accounts for the physical and clinical outcomes observed in sarcopenic adiposity. The greater the combined increase in adiposity and decrease in muscle mass, the worse the outcomes. Created with BioRender.com.

The pathophysiology leading to sarcopenic adiposity/obesity is complex and includes the convergence of aging, hypogonadism, insulin resistance, inflammation, sedentary lifestyle, poor diet quality [178], and fat infiltration, all contributing to the loss of muscle mass, strength, and function. The more detrimental clinical manifestations seen with sarcopenic obesity may simply be that fat mass accrual has exceeded a threshold. As the percentage of total body fat increases, individuals move from a diagnosis of sarcopenia to sarcopenic obesity; however, total body fat may not be a good measure of the fat that has infiltrated muscle [38], although it is a better measure than BMI. Because the term ‘obesity’ is synonymous with BMI, sarcopenic adiposity (SA) would better reflect the pathophysiology of the condition and the associated clinical outcomes. However, unlike sarcopenic obesity, SA is a condition of fat infiltration into muscle, with or without overtly high body fat mass, coupled with muscle mass loss. Muscle functional status can help estimate severity. As total body fat increases, it is logical to assume that muscle fat would also increase; however, this needs to be thoroughly investigated, especially for those with normal-weight obesity. Based on these data, we propose a theoretical model for the developmental stages of sarcopenic adiposity, including its clinical aspects, see Figure 3. Similar effects may be occurring in bone as fat infiltration increases.

**Figure 4:**
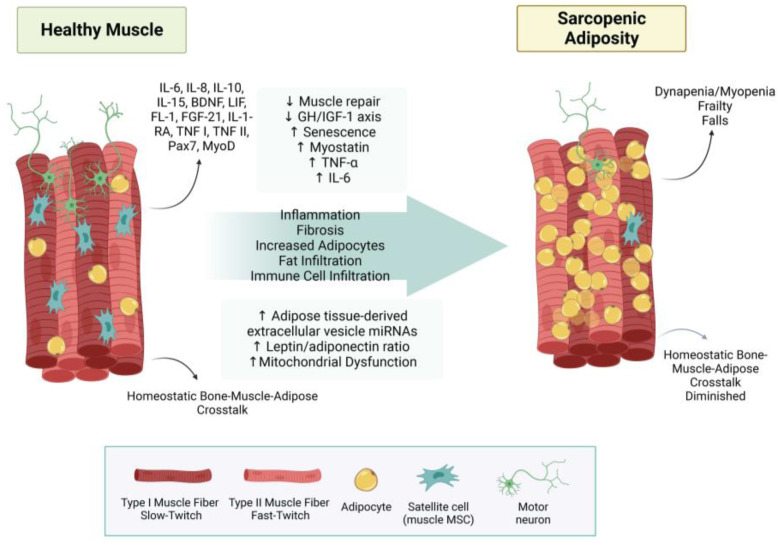
Sarcopenic adiposity syndrome. This figure summarizes how healthy muscle develops into sarcopenic adiposity syndrome. Increased fat infiltration, reduced type 2 fibers, and less innervation of motor neurons. Abbreviations: (↓ = decreased; ↑ = increased; IL-6 = interleukin 6; IL-8 = interleukin 8; IL-10 = interleukin 10; IL-15 = interleukin 15; BDNF = brain-derived neurotrophic factor; LIF = leukemia inhibitory factor; FL-1 = follistatin-like 1; FGF-21 = fibroblast growth factor-21; IL1-RA = interleukin-1 receptor antagonist protein; TNF I = tumor necrosis factor I; TNF II = tumor necrosis factor II; Pax7 = Paired Box 7; MyoD = myoblast determination protein 1; GH/IGF-1 axis = growth hormone/insulin-like growth factor-1 axis; TNF-α = tumor necrosis factor-alpha; miRNA = micro RNA; MSC = mesenchymal stem cell.

Increased levels of inflammatory adipokines contribute to sarcopenia, which is well documented [179,180]. A disruption of myokines necessary for muscle tissue formation and regeneration is also implicated in the pathogenesis of sarcopenia. One group noted an upregulation of tumor necrosis factor-α (TNF-α), interleukin-6 (IL-6), leptin, and myostatin, and a downregulation of interleukin-15 (IL-15) in sarcopenic obesity/adiposity [181]. IL-15 downregulation may be a crucial myokine marker for sarcopenic adiposity. IL-15 maintains immune function, stimulating myogenesis and reducing adipose distribution [172]. It also increases protein accretion, decreases protein degradation, induces myoblast differentiation, increases glucose uptake by skeletal muscle, and plays a role in skeletal muscle hypertrophy, as well as exerting a catabolic effect on adipose tissue, thereby reducing adiposity and regulating body composition [180,182]. Impaired IL-15 signaling may contribute to the coalescence of sarcopenia and obesity. Rodent model studies have shown a decline in IL-15 associated with age and an increase in IL-15 in models displaying higher bone mineral density, reinforcing that tissue cross-talk may be critical for maintaining body composition [180,183]. Concomitantly, the results from an outpatient study with 160 participants found a correlation between low levels of plasma IL-15 and sarcopenia [184]. There also seems to be a higher adiponectin level associated with sarcopenia [185]; however, lower levels are observed in obesity [186]. This suggests adiponectin resistance or localized adiponectin levels are more important than systemic levels. One in vitro study found an activation of muscle regeneration pathways during healthy aging but also indicated evidence of dysregulation in sarcopenia [187]. SA processes are summarized in Figure 4.

### 4.3. Osteosarcopenic Adiposity

The most adverse fate of bone, muscle, and adipose tissue is osteosarcopenic adiposity (OSA), a simultaneous deterioration of bone and muscle, and increased adipose tissue. While the term adiposity may include a high BMI, it more specifically refers to the localized increases in adipose tissue within the bone and muscle and the hypertrophy of adipocytes in white adipose tissue [188]. This illustrates the role of ectopic and ‘hidden’ fat in the constellation of effects present rather than the classical concept of a high BMI. Fabbri et al. found that baseline BMI was not a good predictor of muscle quality (ratio of strength to the cross-sectional area; Nm/cm^2^) in later life [189]. However, those with a higher total fat mass and/or a lower total lean mass at baseline had a higher decline in muscle quality over time. Obese states and the locations of infiltrated fat, create greater risk factors for adverse events due to the reduction of muscle and bone mass, and increased pro-inflammatory cytokines and other endocrine mediators impairing bone and muscle [2]. The terms osteo and sarco in OSA refer to any reduction in bone mass or bone formation (osteopenia, osteoporosis, and any imbalance in bone formation markers) and reduced muscle mass, strength, and function, respectively. Both osteopenia and osteoporosis have established diagnostic criteria. However, diagnosing a reduction in bone formation or detecting other subtle changes in bone and muscle health is more complex. The same difficulty applies to detecting minor changes in adipose tissues and subtle dysfunction in adipocyte metabolism and bone–muscle–fat cross-talk. Something to keep in mind at this point is whether or not osteosarcopenia is a separate entity from osteosarcopenic adiposity; does osteosarcopenia exist without infiltrated fat in both bone and muscle?

Current evidence suggests a shared pathophysiological link between low bone mass, low muscle mass, and an overabundance of fat mass. Osteopenia/osteoporosis, sarcopenia, and overweight/obesity were once considered separate clinical conditions and rarely studied together [12]. Contemporary advances in body composition research point to sufficient evidence to establish a pathophysiological basis for OSA syndrome via bone–muscle–fat cross-talk [3]. However, it is more challenging to establish the co-development of OSA’s components and establish what cause and effect is. For example, Cacciatore et al. recognized that the quality of life domains most affected by OSA are social isolation, frailty, anxiety, disability, depression, and low economic status [43]. It is possible that these domains also contribute to OSA development. In any case, the OSA concept highlights the pressing need to treat diseases and conditions from a whole-body perspective and not in isolation, and in that context, recognizing and treating adiposity is fundamental to OSA. 

All the individual components of OSA are recognized clinical entities, as are sarcopenic obesity and osteosarcopenia. It seems logical to hypothesize that outcomes would worsen if bone and muscle mass loss are combined with adiposity, and newer data are beginning to provide overwhelming support for such assumptions, for example [190,191,192,193]. It is estimated that OSA affects 10% of the adult population 58 years or older globally, and OSA is more likely in older females [194]. In nursing homes, approximately 70% of women and 50% of men were diagnosed with OSA, of which up to one-third were either at risk of malnutrition or were diagnosed as malnourished [195], suggesting poor nutrition is a risk factor for OSA. 

In a cross-sectional study, OSA was associated with worse lung function in Korean adults 50 years and older, without lung disease, compared to those with only one or two components of OSA [196]. This, coupled with data from a Chinese study (mean age 69 years) showing worse scores for gait speed and timed get up and go in those with OSA [197] and others [190,198], suggests OSA has a negative effect on physical performance. Dyslipidemia (presence of one or more of the following: hypercholesterolemia, hypertriglyceridemia, low HDL, or high LDL) was associated with OSA in indigenous ethnic groups in Guangxi, China [199]. Individuals with alcohol disorders are also at a higher risk of OSA than those without [200]. OSA may also increase the risk of falls [201]. In relation to those with a diagnosed disease, breast and prostate cancer patients on hormone depravation therapies may develop OSA, and this deserves research attention to help improve health outcomes in these patients [202]. All these data suggest that OSA is important to diagnose, prevent, and treat.

## 5. Discussion and Conclusions

The concept of OSA syndrome was proposed in 2014 [3]. Each year, more data are added to the literature, adding to the totality of evidence for OSA. OSA includes the option that one condition may have systemic effects that lead to the development of another, through bone–muscle–adipose cross-talk. OSA, therefore, may begin in any one of the three tissues or wherever the adiposity is the most intense. Adipose tissue (adiposity) dysregulation is at the forefront of bone and/or muscle loss, and the eventual development of osteosarcopenic adiposity syndrome is the most serious deterioration in body composition. The prevalence of osteopenic adiposity, sarcopenic adiposity, osteosarcopenia, and osteosarcopenic adiposity are sufficient to garner research attention. In this regard, the need to measure fat mass becomes more apparent and necessary, as does the development of technology for detecting ectopic and redistributed fat. All the evidence suggests that maintaining a healthy fat-mass-to-lean-mass ratio is important for bone and muscle health throughout life. Significant gaps in the literature remain; most notably, more studies in males and younger age groups are needed. On the other hand, the studies are global but use different diagnostic criteria or sometimes the same criteria in individuals of different ethnicities [4,12]. 

One goal of this review was to delineate ‘obesity’ and ‘adiposity’. The latter refers to excess fat mass, fat infiltration into muscle and bone, and the changes to adipose tissue in adiposity. This provides an opportunity to reanalyze any available data sets where body composition measures were expressed in lieu of BMI to provide more insights—a great incentive for further research. New studies in obesity need to include body composition analysis incorporating bone adiposity, muscle adiposity, other ectopic fat, subcutaneous fat, and the pattern of fat redistribution. These studies will help understand the role of fat mass in aging and chronic diseases. It is important to note that technological improvements for assessing fat mass are also required.

Although in the present weight-centered healthcare paradigm [203], measuring fat mass should be a high priority, it is not the case in practice. Regardless, regular assessment of body composition would help diagnose OA, SA, and OSA, possibly at earlier stages, which could result in better overall patient health and quality of life. Obesity is still synonymous with BMI, and this has impeded the widespread use of body composition assessment. BMI has effectively created normal weight obesity or other impaired body composition states, such as metabolic healthy obese, that BMI could not delineate or even explain. A fundamental issue with body composition is the perception that it cannot be measured in the clinical setting and on a mass scale comparable to the measurements of BMI. While free access to MRI, DXA, and other imaging techniques for the purpose of assessing body composition seems outlandish in the current healthcare climate, BIA offers an inexpensive alternative that could easily be used in a clinician’s office. Some newer bioelectrical impedance devices offer an estimate of bone mass (for a description, see [4,12]). Longitudinal use of bioelectrical impedance could help the clinician better assess changes in body composition. 

Fat infiltration clearly plays a role in the loss of bone and/or muscle mass, although it is harder to detect or prove in the clinical setting. Unfortunately, prevention and treatment strategies cannot be implemented without proper diagnosis and the detection of infiltrated fat. Developing techniques will require a large effort from multidisciplinary teams. Nevertheless, the effects of current weight loss interventions (obesity), ranging from bariatric surgery and liposuction, to medications to lifestyle and behavioral interventions, on the numerous fat tissue locales is unknown; this information gap must be addressed. In addition, treatment strategies aimed at SA seem to be focused only on muscle mass [204]. Treatments for OSA have been proposed to maintain bone and muscle mass and reduce fat mass [12], recognizing that all three need to be addressed simultaneously. Furthermore, nutritional studies are now focusing on the role of diet in preventing osteosarcopenia with an emphasis on protein intake and vitamins and minerals such as vitamin D, calcium, and magnesium [44,205,206,207,208]. Nevertheless, in the case of OSA, dietary patterns and diet quality should be considered. Weight loss is usually contraindicated in older adults since studies have suggested that exercise has a minimal osteoprotective effect to mitigate energy restriction-induced bone loss [209]. Therefore, nutritional interventions focusing on diet quality combined with exercise are warranted to determine if this strategy can help older adults offset OSA. Prevention strategies must start early to achieve peak bone mass, achieve and maintain peak muscle mass, and minimize adipose tissue expansion and dysfunction. Early prevention may be more important, considering obesity is occurring at an earlier age—in such cases, the biochemical and physical implications for bone and muscle are unknown. This is a significant gap that requires attention, as early detection of adiposity and bone and muscle health will help people achieve healthier lives. Furthermore, the role of fat infiltration on neurological aspects of bone (bone–brain axis [210]) and muscle loss (motor unit loss [211] and possibly the muscle–brain axis [212]) is needed.

Cross-talk between bone, muscle, and fat exists; however, it is not clear what levels of osteokines, myokines, and adipokines constitute homeostatic cross-talk compared to disrupted cross-talk. One hypothesis derived from this review is that homeostatic cross-talk may be absent in OA, SA, and OSA (bones, muscle, and fat do not talk to each other), causing cellular stress. The changes in muscle fiber types in sarcopenia suggest there may be fiber-specific bone–muscle–fat cross-talk between. The reduced cross-talk between fast twitch fibers and bone and adipose tissues may provide a potential mechanism. Less-known molecular signals may be important in the development of OSA. The inflammatory adipokine chemerin is strongly implicated in OA; however, its role in SA and OSA must be investigated. Ebf1 is an adipose tissue transcription factor that reciprocally regulates the bone–fat switch; however, its actions may be tissue-dependent. As such, it is implicated in OA. However, little is known about its expression in BMAT and local adipocytes in muscle. BMAT produces adiponectin locally, suggesting that fat infiltrated into muscle may also produce adiponectin. This suggests that systemic levels may not be as important as local concentrations. Leptin is also important to maintain muscle mass, possibly by inhibiting myostatin. However, overall, questions remain regarding adipokine production of infiltrated fat. 

**Figure 5:**
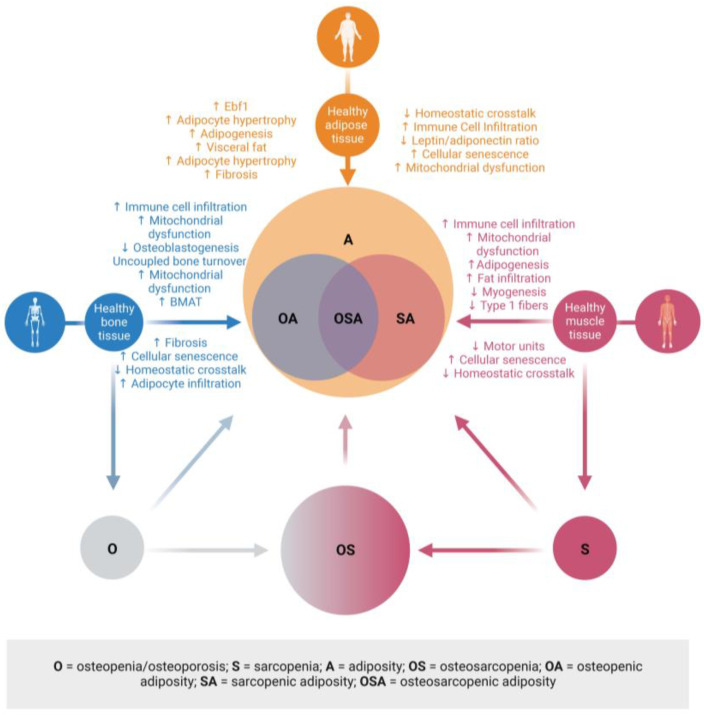
Clinical manifestations of adiposity in bone and fat. The presence of fat in bone and muscle is a key contributor to the pathophysiology and clinical diagnoses of low bone and fat mass and excess fat mass. This new model places adiposity at the center of osteopenic adiposity, sarcopenic adiposity, and osteosarcopenic adiposity. There is the possibility that adiposity, osteoporosis, sarcopenia, and osteosarcopenia can exist independently; therefore, these are also represented. These may also develop into OA, SA, or OSA. Created with BioRender.com Abbreviations: (↓ = decreased; ↑ = increased; Ebf1 = early B-cell factor 1; BMAT = bone marrow adipose tissue. Created with BioRender.com.

Ultimately, it is time to think about each impairment or disease, not existing in isolation but as being interconnected with others and possibly each contributing to the other. That is particularly true for body composition impairments, including OSA [4]. Based on the evidence discussed, a new conceptual model for body composition deterioration is proposed; see Figure 5. This model places adiposity at the center of the development of OSA. It also allows for the existence of osteopenia/osteoporosis, sarcopenia, and osteosarcopenia independent of adiposity, although, at this point, we are not sure whether each can exist without simultaneous fat infiltration. The discussed impairments continuously progress year after year from the very first modification of cross-talk to the most advanced disruption. This creates an opportunity to refine diagnostic criteria further. However, BMI should not be used in any research setting related to bone and muscle loss, as more precise body composition data are needed. Managing adiposity is about managing fat mass, and every effort should be made to precisely measure it. Using the current obesity epidemic as evidence, utilizing proxy fat mass (BMI) measures may not be the best approach. DXA is used to diagnose bone loss and, lately, muscle loss via assessment of appendicular muscle mass. However, DXA is underutilized regarding its use in younger individuals, and clinicians could be more proactive in assessing bone and muscle mass throughout life using BIA. As a final point, learning more about fat mass and its effects on other tissues needs to be the goal of future research in adiposity, as this ignores the critical effects of adipose tissue in the development and clinical presentation of OA, SA, and OSA. 

In conclusion, the role of adipose tissue in bone and muscle health is more complex than previously thought, and this needs to be recognized at the clinical level so that positive changes to the narrative around obesity and body composition can be made. 

## Figures and Tables

**Table 1:** Diagnostic criteria for bone loss, muscle loss, and adiposity in males and females.

Component	Males	Females	Instrument
Bone mass	Bone mineral density T-score ≤ −1.0 standard deviation (SD) from healthy controls at the femoral neck, proximal femur, or lumbar spine		Dual-energy X-ray absorptiometry (DXA)
	Total Bone Mass T-score ≤ −1.0 SD		Bioelectrical Impedance Analysis (BIA)
Muscle mass	Skeletal Mass Index ≤ 5.45 kg/m^−2^	Skeletal Mass Index ≤ 7.26 kg/m^−2^	DXA or BIA
	≤20th percentile of Appendicular Lean Mass		DXA or BIA
	S-Score ≤ −1.0 SD		BIA
Adiposity	Total body fat > 25%	Total body fat > 32%	DXA or BIA
	Fat Mass Index > 9 kg/m^2^	Fat Mass Index > 13 kg/m^2^	DXA or BIA
	Visceral fat > 130 cm^2^	Visceral fat > 110 cm^2^	Computerized Tomography (CT) or Magnetic Resonance Imaging (MRI)
	Visceral/Subcutaneous fat ratio > 1		DXA
	S-Score ≤ −1.0 SD		BIA

## References

[1] JafariNasabian P., Inglis J.E., Reilly W., Kelly O.J., Ilich J.Z. (2017). Aging human body: Changes in bone, muscle and body fat with consequent changes in nutrient intake. J. Endocrinol..

[2] JafariNasabian P., Inglis J.E., Kelly O.J., Ilich J.Z. (2017). Osteosarcopenic obesity in women: Impact, prevalence, and management challenges. Int. J. Women’s Heal..

[3] Ilich J.Z., Kelly O.J., Inglis J.E., Panton L.B., Duque G., Ormsbee M.J. (2014). Interrelationship among muscle, fat, and bone: Connecting the dots on cellular, hormonal, and whole body levels. Ageing Res. Rev..

[4] Ilich J.Z., Gilman J.C., Cvijetic S., Boschiero D. (2020). Chronic Stress Contributes to Osteosarcopenic Adiposity via Inflammation and Immune Modulation: The Case for More Precise Nutritional Investigation. Nutrients.

[5] Ilich J.Z., Kelly O.J., Kim Y., Spicer M.T. (2014). Low-grade chronic inflammation perpetuated by modern diet as a promoter of obesity and osteoporosis. Arch. Ind. Hyg. Toxicol..

[6] Jo A., Mainous A.G. (2018). Informational value of percent body fat with body mass index for the risk of abnormal blood glucose: A nationally representative cross-sectional study. BMJ Open.

[7] Neeland I.J., Ross R., Despres J.P., Matsuzawa Y., Yamashita S., Shai I., Seidell J., Magni P., Santos R.D., Arsenault B. (2019). Visceral and ectopic fat, atherosclerosis, and cardiometabolic disease: A position statement. Lancet Diabetes Endocrinol..

[8] Cunningham S.A., Hardy S.T., Jones R., Ng C., Kramer M.R., Narayan K.M.V. (2022). Changes in the Incidence of Childhood Obesity. Pediatrics.

[9] Romero-Corral A., Somers V.K., Sierra-Johnson J., Korenfeld Y., Boarin S., Korinek J., Jensen M.D., Parati G., Lopez-Jimenez F. (2010). Normal weight obesity: A risk factor for cardiometabolic dysregulation and cardiovascular mortality. Eur. Hear. J..

[10] Franco L.P., Morais C.C., Cominetti C. (2016). Normal-weight obesity syndrome: Diagnosis, prevalence, and clinical implications. Nutr. Rev..

[11] Sahakyan K.R., Somers V.K., Rodriguez-Escudero J.P., Hodge D.O., Carter R.E., Sochor O., Coutinho T., Jensen M.D., Roger V.L., Singh P. (2015). Normal-Weight Central Obesity: Implications for Total and Cardiovascular Mortality. Ann. Intern. Med..

[12] Kelly O.J., Gilman J.C., Boschiero D., Ilich J.Z. (2019). Osteosarcopenic Obesity: Current Knowledge, Revised Identification Criteria and Treatment Principles. Nutrients.

[13] Santilli V., Bernetti A., Mangone M., Paoloni M. (2014). Clinical definition of sarcopenia. Clin. Cases Miner Bone Metab..

[14] Stefanaki C., Peppa M., Boschiero D., Chrousos G.P. (2016). Healthy overweight/obese youth: Early osteosarcopenic obesity features. Eur. J. Clin. Investig..

[15] Salari N., Ghasemi H., Mohammadi L., Behzadi M.H., Rabieenia E., Shohaimi S., Mohammadi M. (2021). The global prevalence of osteoporosis in the world: A comprehensive systematic review and meta-analysis. J. Orthop. Surg. Res..

[16] Singer A., Exuzides A., Spangler L., O’Malley C., Colby C., Johnston K., Agodoa I., Baker J., Kagan R. (2015). Burden of illness for osteoporotic fractures compared with other serious diseases among postmenopausal women in the United States. Mayo Clin. Proc..

[17] Wright N.C., Looker A.C., Saag K.G., Curtis J.R., Delzell E.S., Randall S., Dawson-Hughes B. (2014). The recent prevalence of osteoporosis and low bone mass in the United States based on bone mineral density at the femoral neck or lumbar spine. J. Bone Miner. Res..

[18] Bass M.A., Sharma A., Nahar V.K., Chelf S., Zeller B., Pham L., Allison Ford M. (2019). Bone Mineral Density Among Men and Women Aged 35 to 50 Years. J. Am. Osteopath. Assoc..

[19] Ganji R., Moghbeli M., Sadeghi R., Bayat G., Ganji A. (2019). Prevalence of osteoporosis and osteopenia in men and premenopausal women with celiac disease: A systematic review. Nutr. J..

[20] Qiao D., Liu X., Tu R., Zhang X., Qian X., Zhang H., Jiang J., Tian Z., Wang Y., Dong X. (2020). Gender-specific prevalence and influencing factors of osteopenia and osteoporosis in Chinese rural population: The Henan Rural Cohort Study. BMJ Open.

[21] Petermann-Rocha F., Balntzi V., Gray S.R., Lara J., Ho F.K., Pell J.P., Celis-Morales C. (2022). Global prevalence of sarcopenia and severe sarcopenia: A systematic review and meta-analysis. J. Cachexia Sarcopenia Muscle.

[22] Boutari C., Mantzoros C.S. (2022). A 2022 update on the epidemiology of obesity and a call to action: As its twin COVID-19 pandemic appears to be receding, the obesity and dysmetabolism pandemic continues to rage on. Metabolism.

[23] Ilich J.Z., Kelly O.J., Inglis J.E. (2016). Osteosarcopenic Obesity Syndrome: What Is It and How Can It Be Identified and Diagnosed?. Curr. Gerontol. Geriatr. Res..

[24] Rajan R., Cherian K.E., Kapoor N., Paul T.V. (2020). Trabecular Bone Score-An Emerging Tool in the Management of Osteoporosis. Indian J. Endocrinol. Metab..

[25] Campbell G.M., Sophocleous A. (2014). Quantitative analysis of bone and soft tissue by micro-computed tomography: Applications to ex vivo and in vivo studies. BoneKEy Rep..

[26] Inai R., Nakahara R., Morimitsu Y., Akagi N., Marukawa Y., Matsushita T., Tanaka T., Tada A., Hiraki T., Nasu Y. (2020). Bone microarchitectural analysis using ultra-high-resolution CT in tiger vertebra and human tibia. Eur. Radiol. Exp..

[27] Cruz-Jentoft A.J., Baeyens J.P., Bauer J.M., Boirie Y., Cederholm T., Landi F., Martin F.C., Michel J.P., Rolland Y., Schneider S.M. (2010). Sarcopenia: European consensus on definition and diagnosis: Report of the European Working Group on Sarcopenia in Older People. Age Ageing.

[28] Bhasin S., Travison T.G., Manini T.M., Patel S., Pencina K.M., Fielding R.A., Magaziner J.M., Newman A.B., Kiel D.P., Cooper C. (2020). Sarcopenia Definition: The Position Statements of the Sarcopenia Definition and Outcomes Consortium. J. Am. Geriatr. Soc..

[29] Bluher M. (2020). Metabolically Healthy Obesity. Endocr. Rev..

[30] Tsatsoulis A., Paschou S.A. (2020). Metabolically Healthy Obesity: Criteria, Epidemiology, Controversies, and Consequences. Curr. Obes. Rep..

[31] Elias-Lopez D., Vargas-Vazquez A., Mehta R., Cruz Bautista I., Del Razo Olvera F., Gomez-Velasco D., Almeda Valdes P., Aguilar-Salinas C.A., Metabolic Syndrome Study G. (2021). Natural course of metabolically healthy phenotype and risk of developing Cardiometabolic diseases: A three years follow-up study. BMC Endocr. Disord..

[32] Marques Loureiro L., Lessa S., Mendes R., Pereira S., Saboya C.J., Ramalho A. (2019). Does the Metabolically Healthy Obese Phenotype Protect Adults with Class III Obesity from Biochemical Alterations Related to Bone Metabolism?. Nutrients.

[33] Sukumar D., Becker K.B., Cheung M., Diamond S., Duszak R., Aljahdali A., Volpe S.L., Nasser J.A. (2018). Can bone-regulating hormones and nutrients help characterize the metabolically healthy obese phenotype. Nutr. Health.

[34] Mirzababaei A., Mirzaei K., Khorrami-Nezhad L., Maghbooli Z., Keshavarz S.A. (2017). Metabolically healthy/unhealthy components may modify bone mineral density in obese people. Arch. Osteoporos..

[35] Hunter G.R., Singh H., Carter S.J., Bryan D.R., Fisher G. (2019). Sarcopenia and Its Implications for Metabolic Health. J. Obes..

[36] Tong L.L., Ma X.Y., Tian M., Ding W.Q. (2023). Relationship between skeletal muscle mass index and metabolic phenotypes of obesity in adolescents. Zhongguo Dang Dai Er Ke Za Zhi.

[37] Hwang Y.C., Cho I.J., Jeong I.K., Ahn K.J., Chung H.Y. (2017). Differential association between sarcopenia and metabolic phenotype in Korean young and older adults with and without obesity. Obesity.

[38] Baumgartner R.N. (2000). Body composition in healthy aging. Ann. N. Y. Acad. Sci..

[39] Binkley N., Buehring B. (2009). Beyond FRAX: It’s time to consider “sarco-osteopenia”. J. Clin. Densitom..

[40] Hirschfeld H.P., Kinsella R., Duque G. (2017). Osteosarcopenia: Where bone, muscle, and fat collide. Osteoporos. Int..

[41] Gao Q., Mei F., Shang Y., Hu K., Chen F., Zhao L., Ma B. (2021). Global prevalence of sarcopenic obesity in older adults: A systematic review and meta-analysis. Clin. Nutr..

[42] Kirk B., Zanker J., Duque G. (2020). Osteosarcopenia: Epidemiology, diagnosis, and treatment-facts and numbers. J. Cachexia Sarcopenia Muscle.

[43] Cacciatore S., Massaro C., Landi F. (2023). Preventing Osteoporosis, Sarcopenia and Obesity to Care about Quality of Life. Ann. Geriatr. Med. Res..

[44] Vucic V., Ristic-Medic D., Arsic A., Petrovic S., Paunovic M., Vasiljevic N., Ilich J.Z. (2023). Nutrition and Physical Activity as Modulators of Osteosarcopenic Adiposity: A Scoping Review and Recommendations for Future Research. Nutrients.

[45] Camastra S., Ferrannini E. (2022). Role of anatomical location, cellular phenotype and perfusion of adipose tissue in intermediary metabolism: A narrative review. Rev. Endocr. Metab. Disord..

[46] Fuster J.J., Ouchi N., Gokce N., Walsh K. (2016). Obesity-Induced Changes in Adipose Tissue Microenvironment and Their Impact on Cardiovascular Disease. Circ. Res..

[47] Koenen M., Hill M.A., Cohen P., Sowers J.R. (2021). Obesity, Adipose Tissue and Vascular Dysfunction. Circ. Res..

[48] Sam S., Mazzone T. (2014). Adipose tissue changes in obesity and the impact on metabolic function. Transl. Res..

[49] Kirk B., Feehan J., Lombardi G., Duque G. (2020). Muscle, Bone, and Fat Crosstalk: The Biological Role of Myokines, Osteokines, and Adipokines. Curr. Osteoporos. Rep..

[50] Zhang Y., Zhang C., Wang J., Liu H., Wang M. (2021). Bone-Adipose Tissue Crosstalk: Role of Adipose Tissue Derived Extracellular Vesicles in Bone Diseases. J. Cell. Physiol..

[51] Castano C., Kalko S., Novials A., Parrizas M. (2018). Obesity-associated exosomal miRNAs modulate glucose and lipid metabolism in mice. Proc. Natl. Acad. Sci. USA.

[52] Zong Q., Bundkirchen K., Neunaber C., Noack S. (2023). Are the Properties of Bone Marrow-Derived Mesenchymal Stem Cells Influenced by Overweight and Obesity?. Int. J. Mol. Sci..

[53] Stanford K.I., Goodyear L.J. (2018). Muscle-Adipose Tissue Cross Talk. Cold Spring Harb. Perspect. Med..

[54] Paris M.T., Bell K.E., Mourtzakis M. (2020). Myokines and adipokines in sarcopenia: Understanding cross-talk between skeletal muscle and adipose tissue and the role of exercise. Curr. Opin. Pharmacol..

[55] Rodriguez A., Becerril S., Ezquerro S., Mendez-Gimenez L., Fruhbeck G. (2017). Crosstalk between adipokines and myokines in fat browning. Acta Physiol..

[56] Li F., Li Y., Duan Y., Hu C.A., Tang Y., Yin Y. (2017). Myokines and adipokines: Involvement in the crosstalk between skeletal muscle and adipose tissue. Cytokine Growth Factor Rev..

[57] Leal L.G., Lopes M.A., Batista M.L. (2018). Physical Exercise-Induced Myokines and Muscle-Adipose Tissue Crosstalk: A Review of Current Knowledge and the Implications for Health and Metabolic Diseases. Front. Physiol..

[58] Severinsen M.C.K., Pedersen B.K. (2020). Muscle-Organ Crosstalk: The Emerging Roles of Myokines. Endocr. Rev..

[59] Rao R.R., Long J.Z., White J.P., Svensson K.J., Lou J., Lokurkar I., Jedrychowski M.P., Ruas J.L., Wrann C.D., Lo J.C. (2014). Meteorin-like is a hormone that regulates immune-adipose interactions to increase beige fat thermogenesis. Cell.

[60] He C., He W., Hou J., Chen K., Huang M., Yang M., Luo X., Li C. (2020). Bone and Muscle Crosstalk in Aging. Front. Cell Dev. Biol..

[61] Duque G., Troen B.R. (2022). Osteosarcopenia.

[62] Cariati I., Bonanni R., Onorato F., Mastrogregori A., Rossi D., Iundusi R., Gasbarra E., Tancredi V., Tarantino U. (2021). Role of Physical Activity in Bone-Muscle Crosstalk: Biological Aspects and Clinical Implications. J. Funct. Morphol. Kinesiol..

[63] Yu C., Du Y., Peng Z., Ma C., Fang J., Ma L., Chen F., Zhang C., Geng R., Zhang Y. (2023). Research advances in crosstalk between muscle and bone in osteosarcopenia (Review). Exp. Ther. Med..

[64] Bonewald L. (2019). Use it or lose it to age: A review of bone and muscle communication. Bone.

[65] Paintin J., Cooper C., Dennison E. (2018). Osteosarcopenia. Br. J. Hosp. Med..

[66] Rinonapoli G., Pace V., Ruggiero C., Ceccarini P., Bisaccia M., Meccariello L., Caraffa A. (2021). Obesity and Bone: A Complex Relationship. Int. J. Mol. Sci..

[67] Chen M.B., McAinch A.J., Macaulay S.L., Castelli L.A., O’Brien P E., Dixon J.B., Cameron-Smith D., Kemp B.E., Steinberg G.R. (2005). Impaired activation of AMP-kinase and fatty acid oxidation by globular adiponectin in cultured human skeletal muscle of obese type 2 diabetics. J. Clin. Endocrinol. Metab..

[68] Lewis J.W., Edwards J.R., Naylor A.J., McGettrick H.M. (2021). Adiponectin signalling in bone homeostasis, with age and in disease. Bone Res..

[69] Suzuki S.T., Zhao B., Yang J. (2008). Enhanced muscle by myostatin propeptide increases adipose tissue adiponectin, PPAR-alpha, and PPAR-gamma expressions. Biochem. Biophys. Res. Commun..

[70] Nigro E., Scudiero O., Monaco M.L., Palmieri A., Mazzarella G., Costagliola C., Bianco A., Daniele A. (2014). New insight into adiponectin role in obesity and obesity-related diseases. BioMed Res. Int..

[71] Abou-Samra M., Selvais C.M., Dubuisson N., Brichard S.M. (2020). Adiponectin and Its Mimics on Skeletal Muscle: Insulin Sensitizers, Fat Burners, Exercise Mimickers, Muscling Pills … or Everything Together?. Int. J. Mol. Sci..

[72] Tanaka Y., Kita S., Nishizawa H., Fukuda S., Fujishima Y., Obata Y., Nagao H., Masuda S., Nakamura Y., Shimizu Y. (2019). Adiponectin promotes muscle regeneration through binding to T-cadherin. Sci. Rep..

[73] Raisz L.G., Pilbeam C.C., Fall P.M. (1993). Prostaglandins: Mechanisms of action and regulation of production in bone. Osteoporos. Int..

[74] Upadhyaya S., Kadamkode V., Mahammed R., Doraiswami C., Banerjee G. (2014). Adiponectin and IL-6: Mediators of inflammation in progression of healthy to type 2 diabetes in Indian population. Adipocyte.

[75] Luo X.H., Guo L.J., Xie H., Yuan L.Q., Wu X.P., Zhou H.D., Liao E.Y. (2006). Adiponectin stimulates RANKL and inhibits OPG expression in human osteoblasts through the MAPK signaling pathway. J. Bone Miner. Res..

[76] Krause M.P., Milne K.J., Hawke T.J. (2019). Adiponectin-Consideration for its Role in Skeletal Muscle Health. Int. J. Mol. Sci..

[77] Iwabu M., Yamauchi T., Okada-Iwabu M., Sato K., Nakagawa T., Funata M., Yamaguchi M., Namiki S., Nakayama R., Tabata M. (2010). Adiponectin and AdipoR1 regulate PGC-1alpha and mitochondria by Ca(^2+^) and AMPK/SIRT1. Nature.

[78] Park H.K., Ahima R.S. (2015). Physiology of leptin: Energy homeostasis, neuroendocrine function and metabolism. Metabolism.

[79] Upadhyay J., Farr O.M., Mantzoros C.S. (2015). The role of leptin in regulating bone metabolism. Metabolism.

[80] Dessie G., Ayelign B., Akalu Y., Shibabaw T., Molla M.D. (2021). Effect of Leptin on Chronic Inflammatory Disorders: Insights to Therapeutic Target to Prevent Further Cardiovascular Complication. Diabetes Metab. Syndr. Obes..

[81] Reid I.R., Baldock P.A., Cornish J. (2018). Effects of Leptin on the Skeleton. Endocr. Rev..

[82] Arounleut P., Bowser M., Upadhyay S., Shi X.M., Fulzele S., Johnson M.H., Stranahan A.M., Hill W.D., Isales C.M., Hamrick M.W. (2013). Absence of functional leptin receptor isoforms in the POUND (Lepr(db/lb)) mouse is associated with muscle atrophy and altered myoblast proliferation and differentiation. PLoS ONE.

[83] Batsis J.A., Villareal D.T. (2018). Sarcopenic obesity in older adults: Aetiology, epidemiology and treatment strategies. Nat. Rev. Endocrinol..

[84] Alizadeh Pahlavani H. (2022). Exercise Therapy for People With Sarcopenic Obesity: Myokines and Adipokines as Effective Actors. Front. Endocrinol..

[85] Priego T., Martin A.I., Gonzalez-Hedstrom D., Granado M., Lopez-Calderon A. (2021). Role of hormones in sarcopenia. Vitam. Horm..

[86] Minokoshi Y., Kim Y.B., Peroni O.D., Fryer L.G., Muller C., Carling D., Kahn B.B. (2002). Leptin stimulates fatty-acid oxidation by activating AMP-activated protein kinase. Nature.

[87] Confavreux C.B., Levine R.L., Karsenty G. (2009). A paradigm of integrative physiology, the crosstalk between bone and energy metabolisms. Mol. Cell. Endocrinol..

[88] Kennedy A.J., Davenport A.P. (2018). International Union of Basic and Clinical Pharmacology CIII: Chemerin Receptors CMKLR1 (Chemerin(1)) and GPR1 (Chemerin(2)) Nomenclature, Pharmacology, and Function. Pharmacol. Rev..

[89] Helfer G., Wu Q.F. (2018). Chemerin: A multifaceted adipokine involved in metabolic disorders. J. Endocrinol..

[90] Han L., Zhang Y., Wan S., Wei Q., Shang W., Huang G., Fang P., Min W. (2021). Loss of chemerin triggers bone remodeling in vivo and in vitro. Mol. Metab..

[91] Li J., Zhang T., Huang C., Xu M., Xie W., Pei Q., Xie X., Wang B., Li X. (2021). Chemerin located in bone marrow promotes osteogenic differentiation and bone formation via Akt/Gsk3beta/beta-catenin axis in mice. J. Cell. Physiol..

[92] Shi L., Mao C., Wang X., Liu R., Li L., Mou X., Xu P., Li H., Xu C., Yuan G. (2016). Association of chemerin levels and bone mineral density in Chinese obese postmenopausal women. Medicine.

[93] Jiang X.Y., Wang Q., Zhang Y., Chen Y., Wu L.F. (2022). Association of High Serum Chemerin with Bone Mineral Density Loss and Osteoporotic Fracture in Elderly Chinese Women. Int. J. Women’s Health.

[94] Zhao H., Yan D., Xiang L., Huang C., Li J., Yu X., Huang B., Wang B., Chen J., Xiao T. (2019). Chemokine-like receptor 1 deficiency leads to lower bone mass in male mice. Cell. Mol. Life Sci..

[95] Muruganandan S., Roman A.A., Sinal C.J. (2010). Role of chemerin/CMKLR1 signaling in adipogenesis and osteoblastogenesis of bone marrow stem cells. J. Bone Miner. Res..

[96] Martensson U.E., Bristulf J., Owman C., Olde B. (2005). The mouse chemerin receptor gene, mcmklr1, utilizes alternative promoters for transcription and is regulated by all-trans retinoic acid. Gene.

[97] Xie Q., Deng Y., Huang C., Liu P., Yang Y., Shen W., Gao P. (2015). Chemerin-induced mitochondrial dysfunction in skeletal muscle. J. Cell. Mol. Med..

[98] Sell H., Laurencikiene J., Taube A., Eckardt K., Cramer A., Horrighs A., Arner P., Eckel J. (2009). Chemerin is a novel adipocyte-derived factor inducing insulin resistance in primary human skeletal muscle cells. Diabetes.

[99] Issa M.E., Muruganandan S., Ernst M.C., Parlee S.D., Zabel B.A., Butcher E.C., Sinal C.J., Goralski K.B. (2012). Chemokine-like receptor 1 regulates skeletal muscle cell myogenesis. Am. J. Physiol. Cell Physiol..

[100] Jimenez M.A., Akerblad P., Sigvardsson M., Rosen E.D. (2007). Critical role for Ebf1 and Ebf2 in the adipogenic transcriptional cascade. Mol. Cell. Biol..

[101] Hesslein D.G., Fretz J.A., Xi Y., Nelson T., Zhou S., Lorenzo J.A., Schatz D.G., Horowitz M.C. (2009). Ebf1-dependent control of the osteoblast and adipocyte lineages. Bone.

[102] Fretz J.A., Nelson T., Xi Y., Adams D.J., Rosen C.J., Horowitz M.C. (2010). Altered metabolism and lipodystrophy in the early B-cell factor 1-deficient mouse. Endocrinology.

[103] Li D., Chang X., Connolly J.J., Tian L., Liu Y., Bhoj E.J., Robinson N., Abrams D., Li Y.R., Bradfield J.P. (2017). A genome-wide association study of anorexia nervosa suggests a risk locus implicated in dysregulated leptin signaling. Sci. Rep..

[104] Tsai R.Y., Reed R.R. (1997). Cloning and functional characterization of Roaz, a zinc finger protein that interacts with O/E-1 to regulate gene expression: Implications for olfactory neuronal development. J. Neurosci..

[105] Kang S., Akerblad P., Kiviranta R., Gupta R.K., Kajimura S., Griffin M.J., Min J., Baron R., Rosen E.D. (2012). Regulation of early adipose commitment by Zfp521. PLoS Biol..

[106] Harder L., Puller A.C., Horstmann M.A. (2014). ZNF423: Transcriptional modulation in development and cancer. Mol. Cell. Oncol..

[107] Gao H., Mejhert N., Fretz J.A., Arner E., Lorente-Cebrian S., Ehrlund A., Dahlman-Wright K., Gong X., Stromblad S., Douagi I. (2014). Early B cell factor 1 regulates adipocyte morphology and lipolysis in white adipose tissue. Cell Metab..

[108] Griffin M.J. (2021). Nipping Adipocyte Inflammation in the Bud. Immunometabolism.

[109] Polito A., Barnaba L., Ciarapica D., Azzini E. (2022). Osteosarcopenia: A Narrative Review on Clinical Studies. Int. J. Mol. Sci..

[110] Fassio A., Idolazzi L., Rossini M., Gatti D., Adami G., Giollo A., Viapiana O. (2018). The obesity paradox and osteoporosis. Eat. Weight. Disord..

[111] Liu P.Y., Ilich J.Z., Brummel-Smith K., Ghosh S. (2014). New insight into fat, muscle and bone relationship in women: Determining the threshold at which body fat assumes negative relationship with bone mineral density. Int. J. Prev. Med..

[112] Jiao Y., Sun J., Li Y., Zhao J., Shen J. (2023). Association between Adiposity and Bone Mineral Density in Adults: Insights from a National Survey Analysis. Nutrients.

[113] Bredella M.A., Torriani M., Ghomi R.H., Thomas B.J., Brick D.J., Gerweck A.V., Rosen C.J., Klibanski A., Miller K.K. (2011). Vertebral bone marrow fat is positively associated with visceral fat and inversely associated with IGF-1 in obese women. Obesity.

[114] Kawai M., de Paula F.J., Rosen C.J. (2012). New insights into osteoporosis: The bone-fat connection. J. Intern. Med..

[115] Rosen C.J., Bouxsein M.L. (2006). Mechanisms of disease: Is osteoporosis the obesity of bone?. Nat. Clin. Pract. Rheumatol..

[116] Rosen C.J., Klibanski A. (2009). Bone, fat, and body composition: Evolving concepts in the pathogenesis of osteoporosis. Am. J. Med..

[117] Aaron N., Costa S., Rosen C.J., Qiang L. (2022). The Implications of Bone Marrow Adipose Tissue on Inflammaging. Front. Endocrinol..

[118] Cohen A., Dempster D.W., Stein E.M., Nickolas T.L., Zhou H., McMahon D.J., Muller R., Kohler T., Zwahlen A., Lappe J.M. (2012). Increased marrow adiposity in premenopausal women with idiopathic osteoporosis. J. Clin. Endocrinol. Metab..

[119] Zhong L., Yao L., Seale P., Qin L. (2021). Marrow adipogenic lineage precursor: A new cellular component of marrow adipose tissue. Best Pract. Res. Clin. Endocrinol. Metab..

[120] Li J., Lu L., Liu Y., Yu X. (2022). Bone marrow adiposity during pathologic bone loss: Molecular mechanisms underlying the cellular events. J. Mol. Med..

[121] Farr J.N., Khosla S. (2019). Cellular senescence in bone. Bone.

[122] Matacchione G., Perugini J., Di Mercurio E., Sabbatinelli J., Prattichizzo F., Senzacqua M., Storci G., Dani C., Lezoche G., Guerrieri M. (2022). Senescent macrophages in the human adipose tissue as a source of inflammaging. Geroscience.

[123] Saito Y., Chikenji T.S. (2021). Diverse Roles of Cellular Senescence in Skeletal Muscle Inflammation, Regeneration, and Therapeutics. Front. Pharmacol..

[124] Berryman D.E., Glad C.A., List E.O., Johannsson G. (2013). The GH/IGF-1 axis in obesity: Pathophysiology and therapeutic considerations. Nat. Rev. Endocrinol..

[125] Locatelli V., Bianchi V.E. (2014). Effect of GH/IGF-1 on Bone Metabolism and Osteoporsosis. Int. J. Endocrinol..

[126] Ali D., Tencerova M., Figeac F., Kassem M., Jafari A. (2022). The pathophysiology of osteoporosis in obesity and type 2 diabetes in aging women and men: The mechanisms and roles of increased bone marrow adiposity. Front. Endocrinol..

[127] Korkmaz U., Korkmaz N., Yazici S., Erkan M., Baki A.E., Yazici M., Ozhan H., Ataoglu S. (2012). Anemia as a risk factor for low bone mineral density in postmenopausal Turkish women. Eur. J. Intern. Med..

[128] Marzban M., Nabipour I., Farhadi A., Ostovar A., Larijani B., Darabi A.H., Shabankari E., Gholizade M. (2021). Association between anemia, physical performance and cognitive function in Iranian elderly people: Evidence from Bushehr Elderly Health (BEH) program. BMC Geriatr..

[129] Sebo Z.L., Rendina-Ruedy E., Ables G.P., Lindskog D.M., Rodeheffer M.S., Fazeli P.K., Horowitz M.C. (2019). Bone Marrow Adiposity: Basic and Clinical Implications. Endocr. Rev..

[130] Herrmann M. (2019). Marrow Fat-Secreted Factors as Biomarkers for Osteoporosis. Curr. Osteoporos. Rep..

[131] Styner M., Thompson W.R., Galior K., Uzer G., Wu X., Kadari S., Case N., Xie Z., Sen B., Romaine A. (2014). Bone marrow fat accumulation accelerated by high fat diet is suppressed by exercise. Bone.

[132] Li C.W., Yu K., Shyh-Chang N., Jiang Z., Liu T., Ma S., Luo L., Guang L., Liang K., Ma W. (2022). Pathogenesis of sarcopenia and the relationship with fat mass: Descriptive review. J. Cachexia Sarcopenia Muscle.

[133] Giudice J., Taylor J.M. (2017). Muscle as a paracrine and endocrine organ. Curr. Opin. Pharmacol..

[134] Fonvig C.E., Chabanova E., Ohrt J.D., Nielsen L.A., Pedersen O., Hansen T., Thomsen H.S., Holm J.C. (2015). Multidisciplinary care of obese children and adolescents for one year reduces ectopic fat content in liver and skeletal muscle. BMC Pediatr..

[135] Koster A., Ding J., Stenholm S., Caserotti P., Houston D.K., Nicklas B.J., You T., Lee J.S., Visser M., Newman A.B. (2011). Does the amount of fat mass predict age-related loss of lean mass, muscle strength, and muscle quality in older adults?. J. Gerontol. A Biol. Sci. Med. Sci..

[136] Correa-de-Araujo R., Addison O., Miljkovic I., Goodpaster B.H., Bergman B.C., Clark R.V., Elena J.W., Esser K.A., Ferrucci L., Harris-Love M.O. (2020). Myosteatosis in the Context of Skeletal Muscle Function Deficit: An Interdisciplinary Workshop at the National Institute on Aging. Front. Physiol..

[137] Rosenberg I.H. (1997). Sarcopenia: Origins and clinical relevance. J. Nutr..

[138] Zhou R., Guo Q., Xiao Y., Guo Q., Huang Y., Li C., Luo X. (2021). Endocrine role of bone in the regulation of energy metabolism. Bone Res..

[139] Heinonen S., Jokinen R., Rissanen A., Pietilainen K.H. (2020). White adipose tissue mitochondrial metabolism in health and in obesity. Obes. Rev..

[140] Sabaratnam R., Hansen D.R., Svenningsen P. (2023). White adipose tissue mitochondrial bioenergetics in metabolic diseases. Rev. Endocr. Metab. Disord..

[141] Perez-Rodriguez M., Huertas J.R., Villalba J.M., Casuso R.A. (2023). Mitochondrial adaptations to calorie restriction and bariatric surgery in human skeletal muscle: A systematic review with meta-analysis. Metabolism.

[142] Zalesin K.C., Franklin B.A., Lillystone M.A., Shamoun T., Krause K.R., Chengelis D.L., Mucci S.J., Shaheen K.W., McCullough P.A. (2010). Differential loss of fat and lean mass in the morbidly obese after bariatric surgery. Metab. Syndr. Relat. Disord..

[143] Strain G.W., Gagner M., Pomp A., Dakin G., Inabnet W.B., Hsieh J., Heacock L., Christos P. (2009). Comparison of weight loss and body composition changes with four surgical procedures. Surg. Obes. Relat. Dis..

[144] Huettner F., Rammos C.K., Dynda D.I., Lange M.L., Marshall J.S., Rossi T.R., DeBord J.R. (2012). Body composition analysis in bariatric surgery: Use of air displacement plethysmograph. Am. Surg..

[145] Wu F.Z., Huang Y.L., Wu C.C., Wang Y.C., Pan H.J., Huang C.K., Yeh L.R., Wu M.T. (2016). Differential Effects of Bariatric Surgery Versus Exercise on Excessive Visceral Fat Deposits. Medicine.

[146] Talman A.H., Psaltis P.J., Cameron J.D., Meredith I.T., Seneviratne S.K., Wong D.T. (2014). Epicardial adipose tissue: Far more than a fat depot. Cardiovasc. Diagn. Ther..

[147] Dobson P.F., Dennis E.P., Hipps D., Reeve A., Laude A., Bradshaw C., Stamp C., Smith A., Deehan D.J., Turnbull D.M. (2020). Mitochondrial dysfunction impairs osteogenesis, increases osteoclast activity, and accelerates age related bone loss. Sci. Rep..

[148] Rautenberg E.K., Hamzaoui Y., Coletta D.K. (2022). Mini-review: Mitochondrial DNA methylation in type 2 diabetes and obesity. Front. Endocrinol..

[149] Yan C., Shi Y., Yuan L., Lv D., Sun B., Wang J., Liu X., An F. (2023). Mitochondrial quality control and its role in osteoporosis. Front. Endocrinol..

[150] Wang S., Deng Z., Ma Y., Jin J., Qi F., Li S., Liu C., Lyu F.J., Zheng Q. (2020). The Role of Autophagy and Mitophagy in Bone Metabolic Disorders. Int. J. Biol. Sci..

[151] Liu D., Fan Y.B., Tao X.H., Pan W.L., Wu Y.X., Wang X.H., He Y.Q., Xiao W.F., Li Y.S. (2021). Mitochondrial Quality Control in Sarcopenia: Updated Overview of Mechanisms and Interventions. Aging Dis..

[152] Tian X., Lou S., Shi R. (2023). From mitochondria to sarcopenia: Role of 17beta-estradiol and testosterone. Front. Endocrinol..

[153] Turkel I., Ozerklig B., Yilmaz M., Ulger O., Kubat G.B., Tuncer M. (2023). Mitochondrial transplantation as a possible therapeutic option for sarcopenia. J. Mol. Med..

[154] Ferri E., Marzetti E., Calvani R., Picca A., Cesari M., Arosio B. (2020). Role of Age-Related Mitochondrial Dysfunction in Sarcopenia. Int. J. Mol. Sci..

[155] DeBari M.K., Abbott R.D. (2020). Adipose Tissue Fibrosis: Mechanisms, Models, and Importance. Int. J. Mol. Sci..

[156] Marcelin G., Gautier E.L., Clement K. (2022). Adipose Tissue Fibrosis in Obesity: Etiology and Challenges. Annu. Rev. Physiol..

[157] Musale V., Wasserman D.H., Kang L. (2023). Extracellular matrix remodelling in obesity and metabolic disorders. Life Metab..

[158] Lin X., Patil S., Gao Y.G., Qian A. (2020). The Bone Extracellular Matrix in Bone Formation and Regeneration. Front. Pharmacol..

[159] Alcorta-Sevillano N., Macias I., Infante A., Rodriguez C.I. (2020). Deciphering the Relevance of Bone ECM Signaling. Cells.

[160] Melouane A., Yoshioka M., St-Amand J. (2020). Extracellular matrix/mitochondria pathway: A novel potential target for sarcopenia. Mitochondrion.

[161] Cai L., Shi L., Peng Z., Sun Y., Chen J. (2023). Ageing of skeletal muscle extracellular matrix and mitochondria: Finding a potential link. Ann. Med..

[162] Patel P.S., Buras E.D., Balasubramanyam A. (2013). The role of the immune system in obesity and insulin resistance. J. Obes..

[163] Blaszczak A.M., Jalilvand A., Hsueh W.A. (2021). Adipocytes, Innate Immunity and Obesity: A Mini-Review. Front. Immunol..

[164] Agrawal M., Kern P.A., Nikolajczyk B.S. (2017). The Immune System in Obesity: Developing Paradigms Amidst Inconvenient Truths. Curr. Diab. Rep..

[165] Francisco V., Pino J., Campos-Cabaleiro V., Ruiz-Fernandez C., Mera A., Gonzalez-Gay M.A., Gomez R., Gualillo O. (2018). Obesity, Fat Mass and Immune System: Role for Leptin. Front. Physiol..

[166] Srivastava R.K., Sapra L. (2022). The Rising Era of “Immunoporosis”: Role of Immune System in the Pathophysiology of Osteoporosis. J. Inflamm. Res..

[167] Zhang W., Gao R., Rong X., Zhu S., Cui Y., Liu H., Li M. (2022). Immunoporosis: Role of immune system in the pathophysiology of different types of osteoporosis. Front. Endocrinol..

[168] Ferbebouh M., Vallieres F., Benderdour M., Fernandes J. (2021). The pathophysiology of immunoporosis: Innovative therapeutic targets. Inflamm. Res..

[169] Srivastava R.K., Dar H.Y., Mishra P.K. (2018). Immunoporosis: Immunology of Osteoporosis-Role of T Cells. Front. Immunol..

[170] Zhang X., Li H., He M., Wang J., Wu Y., Li Y. (2022). Immune system and sarcopenia: Presented relationship and future perspective. Exp. Gerontol..

[171] Antuna E., Cachan-Vega C., Bermejo-Millo J.C., Potes Y., Caballero B., Vega-Naredo I., Coto-Montes A., Garcia-Gonzalez C. (2022). Inflammaging: Implications in Sarcopenia. Int. J. Mol. Sci..

[172] Nelke C., Dziewas R., Minnerup J., Meuth S.G., Ruck T. (2019). Skeletal muscle as potential central link between sarcopenia and immune senescence. EBioMedicine.

[173] Wilson D., Jackson T., Sapey E., Lord J.M. (2017). Frailty and sarcopenia: The potential role of an aged immune system. Ageing Res. Rev..

[174] Forte Y.S., Renovato-Martins M., Barja-Fidalgo C. (2023). Cellular and Molecular Mechanisms Associating Obesity to Bone Loss. Cells.

[175] Hou J., He C., He W., Yang M., Luo X., Li C. (2020). Obesity and Bone Health: A Complex Link. Front. Cell. Dev. Biol..

[176] Naot D., Cornish J. (2014). Cytokines and Hormones That Contribute to the Positive Association between Fat and Bone. Front. Endocrinol..

[177] Takeshita S., Fumoto T., Naoe Y., Ikeda K. (2014). Age-related marrow adipogenesis is linked to increased expression of RANKL. J. Biol. Chem..

[178] Goisser S., Kemmler W., Porzel S., Volkert D., Sieber C.C., Bollheimer L.C., Freiberger E. (2015). Sarcopenic obesity and complex interventions with nutrition and exercise in community-dwelling older persons--a narrative review. Clin. Interv. Aging.

[179] Sakuma K., Aoi W., Yamaguchi A. (2015). Current understanding of sarcopenia: Possible candidates modulating muscle mass. Pflug. Arch..

[180] Lutz C.T., Quinn L.S. (2012). Sarcopenia, obesity, and natural killer cell immune senescence in aging: Altered cytokine levels as a common mechanism. Aging.

[181] Vitale G., Cesari M., Mari D. (2016). Aging of the endocrine system and its potential impact on sarcopenia. Eur. J. Intern. Med..

[182] Kalinkovich A., Livshits G. (2017). Sarcopenic obesity or obese sarcopenia: A cross talk between age-associated adipose tissue and skeletal muscle inflammation as a main mechanism of the pathogenesis. Ageing Res. Rev..

[183] Brotto M., Johnson M.L. (2014). Endocrine crosstalk between muscle and bone. Curr. Osteoporos. Rep..

[184] Yalcin A., Silay K., Balik A.R., Avcioğlu G., Aydin A.S. (2018). The relationship between plasma interleukin-15 levels and sarcopenia in outpatient older people. Aging Clin. Exp. Res..

[185] Komici K., Dello Iacono A., De Luca A., Perrotta F., Bencivenga L., Rengo G., Rocca A., Guerra G. (2021). Adiponectin and Sarcopenia: A Systematic Review With Meta-Analysis. Front. Endocrinol..

[186] Achari A.E., Jain S.K. (2017). Adiponectin, a Therapeutic Target for Obesity, Diabetes, and Endothelial Dysfunction. Int. J. Mol. Sci..

[187] Brzeszczynska J., Meyer A., McGregor R., Schilb A., Degen S., Tadini V., Johns N., Langen R., Schols A., Glass D.J. (2018). Alterations in the in vitro and in vivo regulation of muscle regeneration in healthy ageing and the influence of sarcopenia. J. Cachexia Sarcopenia Muscle.

[188] Ilich J.Z., Kelly O.J., Gilman J.C., Cvijetic S., Boschiero D., Duque G., Troen B.R. (2022). Chapter 10—Osteosarcopenic adiposity. Osteosarcopenia.

[189] Fabbri E., Chiles Shaffer N., Gonzalez-Freire M., Shardell M.D., Zoli M., Studenski S.A., Ferrucci L. (2017). Early body composition, but not body mass, is associated with future accelerated decline in muscle quality. J. Cachexia Sarcopenia Muscle.

[190] Sasaki K.I., Kakuma T., Sasaki M., Ishizaki Y., Fukami A., Enomoto M., Adachi H., Matsuse H., Shiba N., Ueno T. (2020). The prevalence of sarcopenia and subtypes in cardiovascular diseases, and a new diagnostic approach. J. Cardiol..

[191] Chen X., Kong C., Yu H., Gong J., Lan L., Zhou L., Gong J., Liu P., Xu L., Deng Q. (2019). Association between osteosarcopenic obesity and hypertension among four minority populations in China: A cross-sectional study. BMJ Open.

[192] Guarnotta V., Prinzi A., Pitrone M., Pizzolanti G., Giordano C. (2020). Circulating Irisin Levels as a Marker of Osteosarcopenic-Obesity in Cushing’s Disease. Diabetes Metab. Syndr. Obes..

[193] Nie Y.Z., Yan Z.Q., Yin H., Shan L.H., Wang J.H., Wu Q.H. (2022). Osteosarcopenic obesity and its components-osteoporosis, sarcopenia, and obesity-are associated with blood cell count-derived inflammation indices in older Chinese people. BMC Geriatr..

[194] Liu Y., Song Y., Hao Q., Wu J. (2023). Global prevalence of osteosarcopenic obesity amongst middle aged and older adults: A systematic review and meta-analysis. Arch. Osteoporos..

[195] Cvijetic S., Keser I., Boschiero D., Ilich J.Z. (2023). Osteosarcopenic Adiposity and Nutritional Status in Older Nursing Home Residents during the COVID-19 Pandemic. Nutrients.

[196] Lim H.S., Kim D.K., Gil H.I., Lee M.Y., Lee H.S., Lee Y.T., Yoon K.J., Park C.H. (2023). Association of Pulmonary Function with Osteosarcopenic Obesity in Older Adults Aged over 50 Years. Nutrients.

[197] Ma Y., Zhang W., Han P., Kohzuki M., Guo Q. (2020). Osteosarcopenic Obesity Associated with Poor Physical Performance in the Elderly Chinese Community. Clin. Interv. Aging.

[198] Szlejf C., Parra-Rodríguez L., Rosas-Carrasco O. (2017). Osteosarcopenic Obesity: Prevalence and Relation With Frailty and Physical Performance in Middle-Aged and Older Women. J. Am. Med. Dir. Assoc..

[199] Mo D., Hsieh P., Yu H., Zhou L., Gong J., Xu L., Liu P., Chen G., Chen Z., Deng Q. (2018). Osteosarcopenic obesity and its relationship with dyslipidemia in women from different ethnic groups of China. Arch. Osteoporos..

[200] Martin-Gonzalez C., Fernandez-Alonso P., Perez-Hernandez O., Abreu-Gonzalez P., Espelosin-Ortega E., Fernandez-Rodriguez C.M., Martin-Ponce E., Gonzalez-Reimers E. (2023). Sarcopenic Obesity in People with Alcoholic Use Disorder: Relation with Inflammation, Vascular Risk Factors and Serum Vitamin D Levels. Int. J. Mol. Sci..

[201] Liu D., Binkley N.C., Perez A., Garrett J.W., Zea R., Summers R.M., Pickhardt P.J. (2023). CT image-based biomarkers acquired by AI-based algorithms for the opportunistic prediction of falls. BJR Open.

[202] Dalla Volta A., Caramella I., Di Mauro P., Bergamini M., Cosentini D., Valcamonico F., Cappelli C., Lagana M., Di Meo N., Farina D. (2023). Role of Body Composition in the Prediction of Skeletal Fragility Induced by Hormone Deprivation Therapies in Cancer Patients. Curr. Oncol. Rep..

[203] O’Hara L., Gregg J. (2006). The war on obesity: A social determinant of health. Health Promot. J. Austr..

[204] Lo J.H.T., Yiu T., Ong M.T.Y., Lee W.Y.W. (2020). Sarcopenia: Current treatments and new regenerative therapeutic approaches. J. Orthop. Translat..

[205] De Rui M., Inelmen E.M., Pigozzo S., Trevisan C., Manzato E., Sergi G. (2019). Dietary strategies for mitigating osteosarcopenia in older adults: A narrative review. Aging Clin. Exp. Res..

[206] Papadopoulou S.K., Papadimitriou K., Voulgaridou G., Georgaki E., Tsotidou E., Zantidou O., Papandreou D. (2021). Exercise and Nutrition Impact on Osteoporosis and Sarcopenia-The Incidence of Osteosarcopenia: A Narrative Review. Nutrients.

[207] Kirk B., Prokopidis K., Duque G. (2021). Nutrients to mitigate osteosarcopenia: The role of protein, vitamin D and calcium. Curr. Opin. Clin. Nutr. Metab. Care.

[208] Ilich J.Z. (2021). Osteosarcopenic adiposity syndrome update and the role of associated minerals and vitamins. Proc. Nutr. Soc..

[209] Wherry S.J., Miller R.M., Jeong S.H., Beavers K.M. (2021). The Ability of Exercise to Mitigate Caloric Restriction-Induced Bone Loss in Older Adults: A Structured Review of RCTs and Narrative Review of Exercise-Induced Changes in Bone Biomarkers. Nutrients.

[210] Schurman C.A., Burton J.B., Rose J., Ellerby L.M., Alliston T., Schilling B. (2023). Molecular and Cellular Crosstalk between Bone and Brain: Accessing Bidirectional Neural and Musculoskeletal Signaling during Aging and Disease. J. Bone Metab..

[211] Machek S.B. (2018). Mechanisms of sarcopenia: Motor unit remodelling and muscle fibre type shifts with ageing. J. Physiol..

[212] Scisciola L., Fontanella R.A., Surina, Cataldo V., Paolisso G., Barbieri M. (2021). Sarcopenia and Cognitive Function: Role of Myokines in Muscle Brain Cross-Talk. Life.

